# Comparative Genomics and Biosynthetic Potential Analysis of Two Lichen-Isolated *Amycolatopsis* Strains

**DOI:** 10.3389/fmicb.2018.00369

**Published:** 2018-03-13

**Authors:** Marina Sánchez-Hidalgo, Ignacio González, Cristian Díaz-Muñoz, Germán Martínez, Olga Genilloud

**Affiliations:** Fundación MEDINA, Centro de Excelencia en Investigación de Medicamentos Innovadores de Andalucía, Granada, Spain

**Keywords:** *Amycolatopsis*, secondary metabolites, phylogeny, whole genome sequence, biosynthetic gene clusters

## Abstract

Actinomycetes have been extensively exploited as one of the most prolific secondary metabolite-producer sources and continue to be in the focus of interest in the constant search of novel bioactive compounds. The availability of less expensive next generation genome sequencing techniques has not only confirmed the extraordinary richness and broad distribution of silent natural product biosynthetic gene clusters among these bacterial genomes, but also has allowed the incorporation of genomics in bacterial taxonomy and systematics. As part of our efforts to isolate novel strains from unique environments, we explored lichen-associated microbial communities as unique assemblages to be studied as potential sources of novel bioactive natural products with application in biotechnology and drug discovery. In this work, we have studied the whole genome sequences of two new *Amycolatopsis* strains (CA-126428 and CA-128772) isolated from tropical lichens, and performed a comparative genomic analysis with 41 publicly available *Amycolatopsis* genomes. This work has not only permitted to infer and discuss their taxonomic position on the basis of the different phylogenetic approaches used, but has also allowed to assess the richness and uniqueness of the biosynthetic pathways associated to primary and secondary metabolism, and to provide a first insight on the potential role of these bacteria in the lichen-associated microbial community.

## Introduction

The class *Actinobacteria* was defined for Gram-positive bacteria with high genomic G+C content (over 55%), among which are included major families of actinomycetes that produce almost 75% of all known secondary metabolites, many of them of high relevance for human health and biotechnology industry (Barka et al., 2016). These compounds have been shown to include a wide range of industrial and medical applications, as drugs (i.e., antifungals, antibacterials, antitumorals, or immunosuppresors), herbicides and plant growth promoting agents among others (Genilloud et al., 2011; Sharma et al., 2014; Genilloud, 2017a). Within the actinomycetes, the genus *Streptomyces* is the most prolific and most studied producer of secondary metabolites, but members of the families *Pseudonocardiaceae* and *Micromonosporaceae* have also shown to produce a broad diversity of bioactive molecules. Among *Pseudonocardiaceae*, members of the genus *Amycolatopsis* produce several relevant secondary metabolites, such as balhimycin, vancomycin, avoparcin, ristomycin, chelocardin, chloroeremomycin, ECO-0501 and rifamycin (Chen et al., 2016; Kumari et al., 2016). More recently, other antibiotics have been described from some *Amycolatopsis* strains such as macrotermycins A–D (Beemelmanns et al., 2017), pargamicins B–D (Hashizume et al., 2017) and rifamorpholines A–E (Xiao et al., 2017). In addition, the importance of *Amycolatopsis* strains in industrial processes such as bioremediation (heavy metal immobilization, herbicide and polymers biodegradation) and bioconversion (wuxistatin and vanillin production) (Dávila Costa and Amoroso, 2014), has been clearly demonstrated.

Members of the genus *Amycolatopsis* were originally misidentified as *Streptomyces*, then as *Nocardia* to be finally recognized as belonging to a new genus with species lacking mycolic acid in their cell wall (Lechevalier et al., 1986). A total of 70 *Amycolatopsis* species have been recognized so far (http://www.bacterio.net/amycolatopsis.html) and isolated from a broad diversity of environments ranging from soils, plants, and ocean sediments to clinical sources. Most species of *Amycolatopsis* belong to two major subclades: *A. methanolica* (AMS) and *A. orientalis* (AOS) (Tang et al., 2016). Several AOS species have been shown to synthesize antibiotics, while AMS strains show a biotechnological potential for the overproduction of aromatic amino acids and bioremediation (Tang et al., 2016).

Recent advances in the field of next generation sequencing (NGS), have allowed an exponential increase of bacterial genome sequences available in public databases, being the genomes of *Streptomyces* spp. the most intensively studied (Loman and Pallen, 2015; Chen et al., 2016; Kumari et al., 2016). In the case of *Amycolatopsis*, as of June 2017, 54 sequencing genome projects had been assembled (www.ncbi.nlm.nih.gov/assembly) from which 30 belong to type strains. The 9 genome projects that have been completely sequenced have shown that *Amycolatopsis* strains contain comparatively large genomes (from 5 to 10 Mb) in the form of a circular chromosome. The analysis of these genomes with improved algorithms has revealed that *Amycolatopsis* strains harbor many more cryptic biosynthetic gene clusters (BGCs) than previously estimated. Eleven BGCs from *Amycolatopsis* have been characterized and compiled in the MIBiG (Minimum Information about a Biosynthetic Gene cluster) Repository (Table 1) (http://mibig.secondarymetabolites.org, Medema et al., 2015). Moreover, this increased availability of new genome sequences in public databases is allowing a deeper characterization of microbial biosynthetic potential using genomic data as well as a new standardization of new taxonomic approaches based on full genome sequence information (Colston et al., 2014).

**Table 1:** Biosynthetic gene clusters from *Amycolatopsis* strains described in the MIBiG database.

**Organism**	**Main product**	**Biosynthetic class**	**MIBiG accession**	**Full/partial MIBiG info**
*A. alba* DSM 44262^T^	Albachelin	NRP	BGC0001211	Partial
*A. balhimycina* DSM 44591^T^	Balhimycin	NRP	BGC0000311	Partial
*A. japonica* DSM 44213^T^	Ristomycin A	NRP/Saccharide	BGC0000419	Full
*A. lurida* DSM 43134^T^	Ristocetin	NRP	BGC0000418	Partial
*A. mediterranei* S699	Rifamycin	Polyketide	BGC0000136	Partial
*A. orientalis* DSM 40040^T^	Quartromicin	Polyketide	BGC0000133	Partial
*A. orientalis* A82846	Chloroeremomycin	NRP	BGC0000322	Partial
*A. orientalis* HCCB10007	Vancomycin	NRP	BGC0000455	Partial
*A. orientalis* subsp. *vinearia* BA-07585	BE-7585A	Polyketide	BGC0000203	Partial
*Amycolatopsis* sp. SANK 60206	A-102395	Other	BGC0001191	Partial
*A. sulphurea* DSM 46092^T^	Chelocardin	Polyketide	BGC0000208	Partial

The emergence of antimicrobial resistance against frequently used antibiotics has brought to light the urgent need of novel antibacterial compounds, and the necessity to look for alternative isolation sources and new drug discovery strategies to identify novel chemical classes of compounds (Adamek et al., 2017). As part of our integrated antibiotic discovery programs, we selected two bioactive *Amycolatopsis* strains (CA-128772 and CA-126428) previously isolated in our laboratory from lichens collected respectively in tropical areas from Hawaii and Reunion islands (González et al., 2005). Lichens are symbiotic associations of a fungal mycobiont, one or more algal or cyanobacterial photobionts and a diverse community of associated microbes, and represent important sources of natural products, mostly produced by the mycobiont. The *Alphaproteobacteria* are the predominant lichen-associated bacteria, but *Actinobacteria, Firmicutes, Betaproteobacteria, Deltaproteobacteria*, and *Gammaproteobacteria* have been also identified (Cardinale et al., 2006; Aschenbrenner et al., 2016). These lichen-associated bacterial communities have been suggested to play important roles in the symbiosis, being of special interest the populations of the orders *Burkholderiales* and *Actinomycetales*, well known as prolific secondary metabolite producers (Calcott et al., 2017). Recent studies have confirmed that lichen-associated bacteria produce new bioactive substances, especially among *Streptomyces* species (González et al., 2005; Cardinale et al., 2006; Parrot et al., 2015; Calcott et al., 2017; Liu et al., 2017). However, few actinomycetes belonging to the genus *Amycolatopsis* have been isolated so far from these sources (González et al., 2005; Liu et al., 2017), what triggered our interest to study their biosynthetic potential and suggest a role in this unique environment.

In this work, we have established the taxonomic position and mined the draft genomes of two new strains of *Amycolatopsis*, CA-126428 and CA-128772. The study has permitted to perform a comparative analysis with all publicly available complete or draft *Amycolatopsis* genomes, with a specific focus on the richness and diversity of their BGCs.

## Materials and methods

### DNA extraction and whole genome next generation sequencing

Genomic DNAs from strains CA-126428 and CA-128772 were extracted and purified as previously described (Kieser et al., 2000) from strains grown in ATCC-2 liquid medium [0.5% yeast extract (Difco, Franklin Lakes, NJ, USA), 0.3% beef extract (Difco), 0.5% peptone (Difco), 0.1% dextrose (Difco), 0.2% starch from potato (Panreac, Barcelona, Spain), 0.1% CaCO3 (E. Merck, Darmstadt, Germany), and 0.5% NZ amine E (Sigma, St Louis, MO, USA)].

Genomes of strains CA-126428 and CA-128772 were sequenced *de novo* by Macrogen (Seoul, Korea; http://www.macrogen.com/) and Service XS (Leiden, the Netherlands; http://www.servicexs.com), respectively, using the Illumina HiSeq 2500 platform. Paired-end libraries were created using the NEBNext Ultra DNA library prep kit (New England Biolabs). The quality and yield after sample preparation was measured with the Fragment Analyzer (AATI), and the size of the resulting product was consistent with the expected size of 500–700 bp. Clustering and DNA sequencing was performed according to manufacturer's protocol. A concentration of 15.0 pM DNA was used. Image analysis, base calling and quality check was performed with the Illumina data analysis pipeline RTA v1.18.64 and Bcl2fastq v2.17. A dataset of at least 1.3 Gb per sample was delivered. Prior to assembly, the reads were trimmed for adapter sequences and filtered for sequence quality. Presumed adapter sequences were removed from the read when the bases matched a sequence in the adapter sequence set (TruSeq adapters) with 2 or less mismatches and an alignment score of at least 12. Bases with phred scores below Q22 were removed from the reads. Glimmer v3.2 (Aggarwal and Ramaswamy, 2002) was used for annotation.

### 16S ribosomal RNA (rRNA) gene amplification and sequencing

Since the sequences of the 16S rRNA genes were incomplete in the genomes, PCR primers FD1 and RP2 were used to amplify the nearly full-length 16S rRNA genes of the strains CA-126428 and CA-128772 (Weisburg et al., 1991). PCR products were sequenced by Secugen (Madrid, Spain; http://www.secugen.es/) with primers FD1, RP2, 1100R, and 926F (Lane, 1991). Partial sequences were assembled and edited using the Assembler contig editor component of Bionumerics 5.10 analysis software (Applied Maths NV, Sint-Martens-Latem, Belgium).

The identification of the closest match sequences was performed at the EzBiocloud server (http://www.ezbiocloud.net/identify) (Yoon et al., 2017).

### Strains and sequences

Strains CA-128772 and CA-126428 belong to MEDINA's microbial collection and were previously isolated from arboricolous lichens collected in humid tropical forests from Hawaii and dry tropical forests from Reunion islands, respectively, as previously described (González et al., 2005). In brief, each lichen sample (300–500 mg) was washed twice with sterile water and homogenized in 30 ml of sterile water using a blender. Serial dilutions were plated on selective actinomycete isolation media (González et al., 2005). Individual colonies were isolated and grown at 28°C on YME agar medium (0.4% yeast extract, 1% malt extract, 0.4% glucose and 0.2% Bacto-agar).

The complete 16S rRNA sequences of 70 *Amycolatopsis* type strains were downloaded from the List of Prokaryotic Names with Standing in Nomenclature (LPSN) database (http://www.bacterio.net/amycolatopsis.html) (Supplementary Table 1). The sequences ranged from 1,351 to 1,530 bp. A set of publicly available 41 complete and draft genomes from *Amycolatopsis* strains and the 16S rRNA gene sequences of *Amycolatopsis* non-type strains were downloaded from NCBI (https://www.ncbi.nlm.nih.gov) (Supplementary Table 1).

### Phylogenetic analysis

#### 16S rRNA analysis

The 16S rRNA sequences were aligned with the MEGA 7.0.26 package (http://www.megasoftware.net) (Kumar et al., 2016) using Clustawl. The pairwise deletion Neighbor-Joining method of the MEGA 7.0.26 package (http://www.megasoftware.net) (Kumar et al., 2016), corrected with Jukes-Cantor algorithms and a bootstrap of 1,000 replicates, was used to construct a phylogenetic tree based on the complete 16S rRNA sequences. *Micromonospora chalcea* DMS 43026^T^ (Foulerton, 1905) 16S rRNA gene was used as outgroup. Genomic distances were calculated with the Kimura-2 parameter model included also in the MEGA software.

#### Multi-locus sequence analysis (MLSA)

Four single-copy housekeeping genes present in all the genomes were used for MLSA analysis: *atpD* (ATP synthase F1, beta subunit), *dnaK* (Hsp70 chaperone), *recA* (recombinase A) and *rpoB* (RNA polymerase beta subunit) (Sentausa and Fournier, 2013; Glaeser and Kämpfer, 2015). The sequences of each of these genes were extracted from the *Amycolatopsis* genomes using the Multi-tBlastN algorithm (https://blast.ncbi.nlm.nih.gov) and were subsequently concatenated with Geneious 9.1.8 software (Biomatters, www.geneious.com, (Kearse et al., 2012), generating a sequence of approximately 7.8 Kb. A phylogenetic tree was constructed using the MEGA 7.0.26. pairwise deletion Neighbor-Joining and Jukes-Cantor algorithms methods and a bootstrap of 1,000 replicates (Kumar et al., 2016). *Micromonospora chalcea* DMS 43026^T^ (Foulerton, 1905) concatenated housekeeping genes were used as outgroup. Genomic distances were calculated with the Kimura-2 parameter model included also in the MEGA software.

### Primary metabolism analysis

The genomes were functionally characterized in the KEGG Database (http://www.kegg.jp) (Kanehisa et al., 2016a). Ortholog K numbers were assigned by the BlastKOALA sequence similarity tool (http://www.kegg.jp/blastkoala/) (Kanehisa et al., 2016b). KEGG mapper tool (http://www.kegg.jp/kegg/tool/map_pathway.html) was used to reconstruct and compare the metabolic pathways.

### Secondary metabolite pathways analysis

The presence of BGCs in the genomes was analyzed using antiSMASH 4.0, (http://antismash.secondarymetabolites.org) (Blin et al., 2017) including Clusterfinder with a minimum probability of 60%.

### Genome comparisons

Genome comparisons were performed on both complete and draft genomes. The contigs of draft genomes were concatenated with Geneious 9.1.8 software (Biomatters, www.geneious.com), (Kearse et al., 2012) to facilitate analysis.

Gene synteny analysis was assessed with progressiveMauve (http://darlinglab.org/mauve/), (Darling et al., 2010). To compare and visualize genomic regions from Mauve results we used the R-package genoPlotR (http://genoplotr.r-forge.r-project.org/) (Guy et al., 2011).

The genome sequence similarity between *Amycolatopsis* strains was evaluated with the Genome-to-Genome Distance Calculator (GGDC) 2.1 online software (http://ggdc.dsmz.de), (Auch et al., 2010; Meier-Kolthoff et al., 2013, 2014).

The Average Nucleotide Identity (ANI) and the orthology of genome sequences were calculated with OrthoANI, (http://www.ezbiocloud.net/sw/oat), (Lee et al., 2016).

## Results and discussion

### Whole genome sequencing

The genomes of strains CA-126428 and CA-128772 were sequenced, assembled and annotated by external providers and their characteristics are summarized in Table 2. A total of 2 × 10,113,591 paired reads for CA-128772 and 2 × 6,420,119 paired reads for CA-126428 were generated with theoretical coverage >100 ×. All reads were assembled to a draft genome of 10.5 Mb for CA-126428 and 10.2 Mb for CA-128772, with a G+C content of 71.4 and 71.8%, respectively. These G+C content values are similar to those of some *Amycolatopsis* complete genomes, such as the strains of *A. mediterranei* U32 and S699 (Kumari et al., 2016). A total of 188 contigs and 9626 coding DNA sequences (CDSs) were obtained for CA-126428, while 121 contigs and 10986 CDSs were assembled for CA-128772. The inconsistency between the number of CDS and the length of the genomes can be explained by the N50 and the average length of the contigs. As it is shown in Table 2, the genome sequence from strain CA-128772 presents a higher N50 value (186,760) and a higher average contig length (83,989 bp) than strain CA-126428 (N50 = 139,227, average length = 55,808 bp) suggesting that the CA-126428 genome is more fragmented and probably more CDSs are missed.

**Table 2:** NGS statistic data of CA-126428 and CA-128772 draft genomes.

**Strain**	**# Contigs**	**Contigs sum (bp)**	**N50**	**Longest contig (bp)**	**Shortest contig (bp)**	**Average length (bp)**	**% GC content**	**# CDS**
CA-126428	188	10,492,049	139,227	724,738	1,147	55,808	71.39	9,626
CA-128772	121	10,162,752	186,760	371,657	231	83,989	71.80	10,896

These Whole Genome Shotgun projects were deposited at DDBJ/ENA/GenBank under the accession numbers PPHF00000000 (strain CA-126428) and PPHG00000000 (strain CA-128772). The versions described in this paper are versions PPHF01000000 and PPHG01000000 (Supplementary Table 1).

### Taxonomic identification and phylogenetic analysis of strains CA-126428 and CA-128772

The molecular identification of strains CA-128772 and CA-126428 was based on the comparison of their 16S rRNA gene sequences to reference type strains sequences using the EzBiocloud server. Both strains showed to contain only one 16S rRNA copy. The 16S rRNA gene sequences were deposited at GenBank under the accession numbers MG800320 (strain CA-126428) and MG799844 (strain CA-128772) (Supplementary Table 1). Strain CA-128772 showed the highest similarity values with *A. pretoriensis* DSM 44654^T^ (99.79%), *A. rifamycinica* DSM 46095^T^ (99.58%), *A. lexingtoniensis* DSM 44653^T^ (99.58%) and *A. tolypomycina* DSM 44544^T^ (99.50%). With respect to isolate CA-126428, the highest similarity values were obtained with *A. mediterranei* DSM 43304^T^ (99.51%), *A. kentuckyensis* DSM 44652^T^ (99.44%), *A. rifamycinica* DSM 46095^T^ (99.44%) and *A. pretoriensis* DSM 44654^T^ (99.37%).

**Figure 1:**
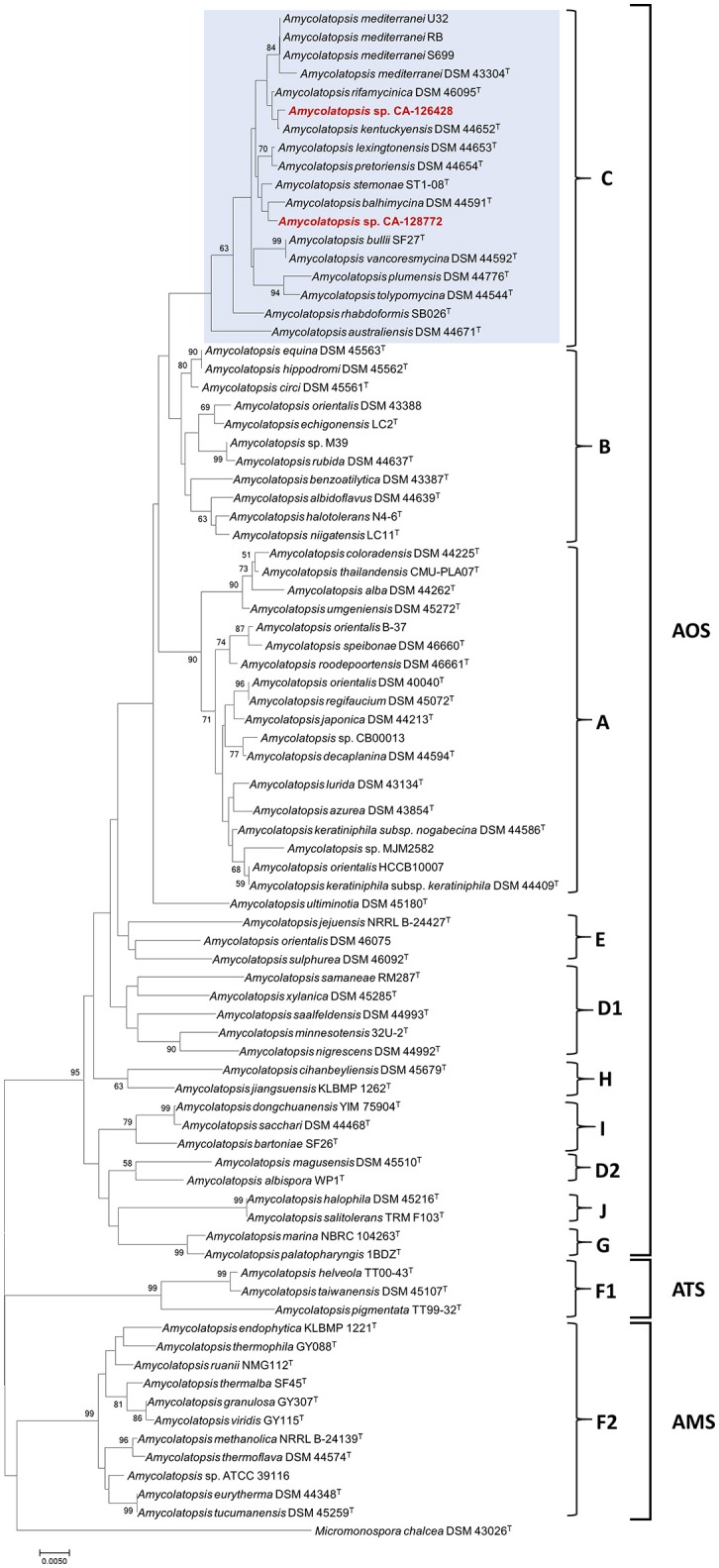
Phylogenetic NJ tree based on 16S rRNA gene sequences from 82 *Amycolatopsis* strains. The scale bar indicates 5 nucleotide substitutions per 1,000 nucleotides, and the node numbers are percentage bootstrap values based on 1,000 resampled datasets. Bootstrap values below 50% are not shown. The AOS, ATS and AMS subclades are indicated, as well as the groups A-J. The strains grouped with CA-126428 and CA-128772 isolates (group C) are blue-shaded.

To confirm the taxonomic assignment and the existing phylogenetic relationships between our isolates and other reference and non-type *Amycolatopsis* strains (Supplementary Table 1), a phylogenetic tree was built based on the complete 16S rRNA gene sequences, using *Micromonospora chalcea* DSM 43026^T^ as outgroup (Figure 1). *Amycolatopsis* species group into three major subclades including the mesophilic or moderately thermophilic *A. orientalis* (AOS) subclade, the thermophilic *A. methanolica* (AMS) subclade, and the mesophilic *A. taiwanensis* (ATS) subclade (Figure 1) (Tang et al., 2016). The AOS subclade includes previously defined groups A-E and G (Everest and Meyers, 2009, 2011) and groups H, I, and J (Tang et al., 2016). The ATS and AMS subclades include the F group (Everest and Meyers, 2009, 2011), that was latter divided in F1 (ATS) and F2 (AMS) (Tang et al., 2016).

The phylogenetic tree associates both strains CA-126428 and CA-128772 to the AOS subclade, containing 36 type species from which three have a full genome sequence, as well as three non-type strains of *A. mediterranei* (RB, S699 and U32), five non-type strains of *A. orientalis* (DSM 43388, DSM 46075, HCCB10007, CB00013, and B-37) and two strains of *Amycolatopsis* sp. (M39 and CB00013). Both CA-126428 and CA-128772 strains, together with the three non-type *A. mediterranei* strains, are included in a branch containing 13 type strains, identified as group C (Figure 1). Within the AMS subclade, another non-type genome-sequenced strain, *Amycolatopsis* sp. ATCCC 39116, was included (Figure 1). In general, the topology of the groups A, B, C, F1, F2, G, H, and I is conserved in our tree despite the additional species that were not previously analyzed (Figure 1). The only exceptions are the group D, that is divided in two groups (D1 and D2), and the strain *A. ultiminotia* DSM 45180^T^, that is no longer included in group E (Figure 1).

The closest genetic distances were determined for strain CA-128772 with *A. pretoriensis* DSM 44654^T^ (0.003) and for strain CA-126428 with *A. kentuckyensis* DSM 44652^T^ (0.001) (Supplementary Figure 1). The distances among the strains in group C ranged between 0.001 and 0.02, and no distance was observed among the non-type *A. mediterranei* strains. Strains *A. helveola* TT00-43^T^, *A. taiwanensis* DSM 45107^T^ and *A. pigmentata* TT99-32^T^ showed the highest evolutionary distances (0.07–0.09) with the rest of strains. Nonetheless, these values are very low and show that these species are very closely-related.

**Figure 2:**
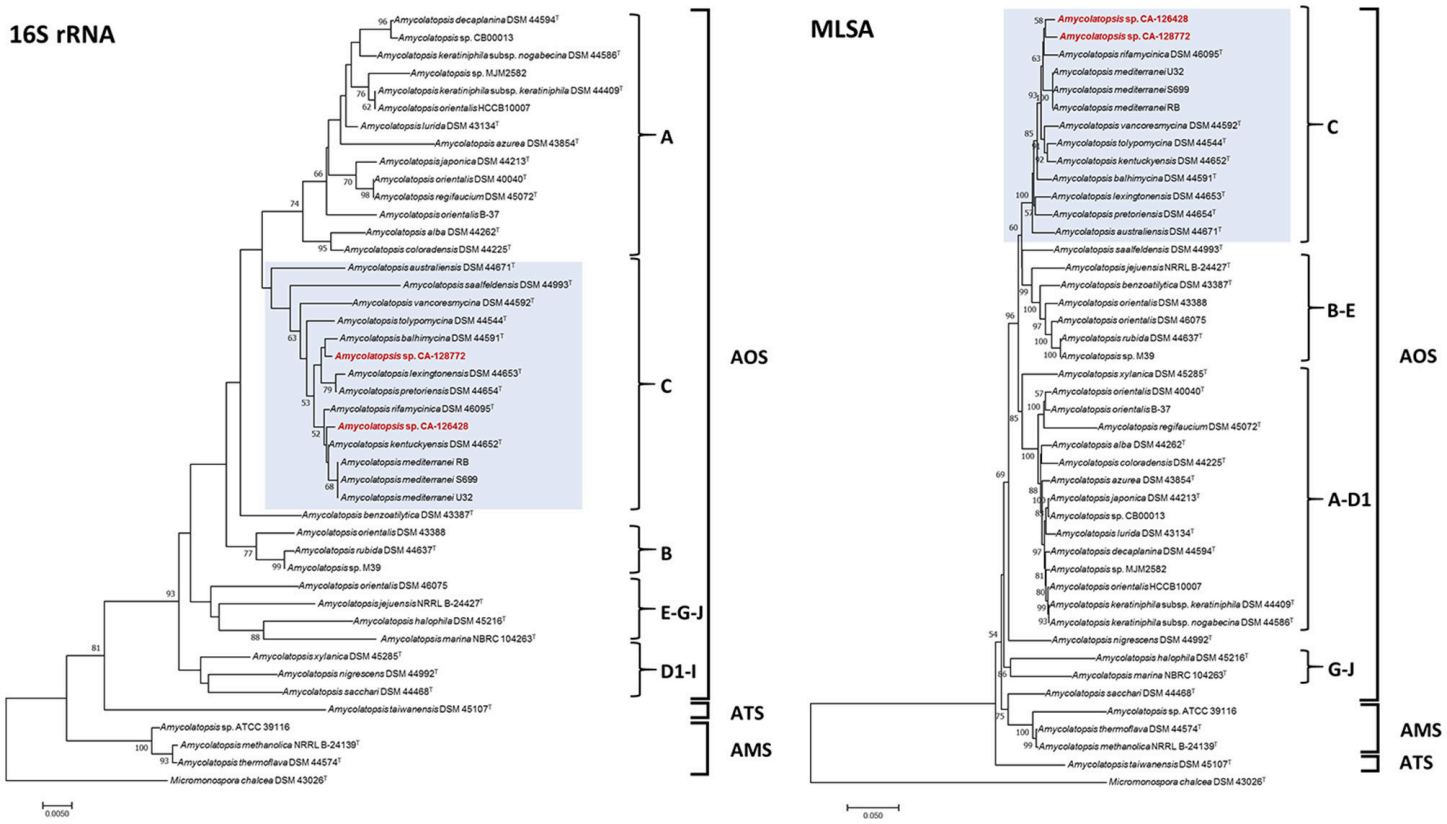
Phylogenetic NJ trees based on 16S rRNA gene sequences **(Left)** and four concatenated sequences (*atpD, dnaK, recA* and *rpoB*) (MLSA, **Right**) from 43 genome-sequenced *Amycolatopsis* strains. The scale bars indicate the percentage of difference in the nucleotide sequences, and the node numbers are percentage bootstrap values based on 1,000 resampled datasets. Bootstrap values below 50% are not shown. The AOS, ATS and AMS subclades are indicated, as well as the groups A–J. The strains grouped with CA-126428 and CA-128772 isolates (group C) are blue-shaded.

Two new 16S rRNA- and MLSA-based phylogenetic trees were constructed only including the 43 genome-sequenced strains (Figure 2). The MLSA tree was constructed from four-gene (*atpD, dnaK, recA*, and *rpoB*) concatenated nucleotide sequences. As shown in Figure 2, the three AOS, ATS and AMS subclades are present in both trees, but some differences are observed in the organization of the groups in AOS subclade. The same A, B and C groups are defined in both trees, whereas in the MLSA tree, group B includes strains from group E and group A includes strains from group D1. In the 16S rRNA-based tree strains from groups E, G, and J clustered together, as well as strains from D1 and I groups, whereas in the MLSA tree, strains from groups G and J are clustered.

**Figure 3:**
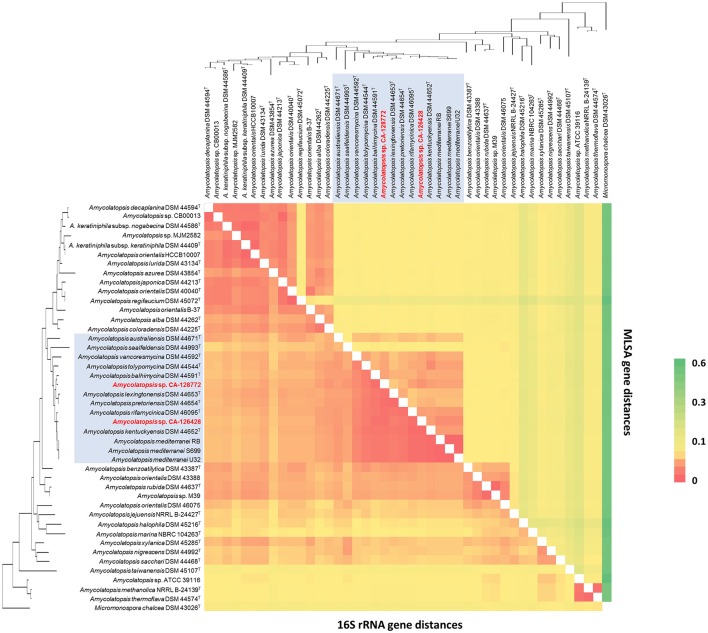
16S rRNA (Bottom left) vs. MLSA (Top right) genomic distances heatmap using the Kimura 2-parameter model. Self-genome comparisons occur on a diagonal line stretching from the top left to the bottom right corners. The strains are ordered in the same way as the 16S phylogenetic tree in Figure 2, which has been placed upon both axes for orientation. The strains belonging to group C have been shaded in blue for clarification. The heatmap legend is shown on the right.

The composition of group C, that contains strains CA-126428 and CA-128772, is conserved in both 16S rRNA and MLSA trees, except for the strain *A. saalfeldensis* DSM 44993^T^, which is not included in the group in the MLSA tree. However, the relative position of the strains differs in both trees. In the 16S rRNA tree strain CA-126428 is closely related to the strains *A. rifamycinica* DSM 46095^T^, *A. kentuckyensis* DSM 44652^T^ and *A. mediterranei*, and strain CA-128772 is closely related to *A. balhimycina* DSM 44591^T^, *A. lexingtoniensis* DSM 44653^T^ and *A. pretoriensis* DSM 44654^T^. In contrast, in the MLSA tree, both strains cluster together and are closely related to *A. rifamycinica* DSM 46095^T^ and the *A. mediterranei* strains. Nevertheless none of these relationships were supported by the bootstrap values, suggesting that both strains could represent novel species within the genus. Bootstrap values of group C (100% in MLSA tree and >50% in 16S rRNA tree) support the significance of the MLSA-based phylogeny. The genomic distances based on both 16S rRNA and MLSA phylogenetic analyses are shown in Figure 3. The 16S rRNA sequence distances ranged from 0 to 0.096 (mean 0.039), while MLSA distances present a broader range between 0 and 0.582 (mean 0.071).

Previous whole genome sequence studies (Tang et al., 2016) have shown that some *Amycolatopsis* strains present multiple copies of 16S rRNA genes, which is not the case of strains CA-126428 and CA-128772. The 16S rRNA sequence identities for some inter-species pairs are higher than those of the corresponding intra-species pairs and this fact may influence the structure of *Amycolatopsis* 16S rRNA gene phylogenetic trees (Tang et al., 2016). The lack of resolution at the species level of 16S rRNA gene-based phylogenies can be overcome by MLSA methods (Glaeser and Kämpfer, 2015). The observed inconsistency between MLSA and 16S rRNA gene phylogenies reinforce the idea that MLSA allows a much more precise species delineation within bacteria (Stackebrandt et al., 2002; Konstantinidis and Tiedje, 2007; Thompson et al., 2013). However, given that not all housekeeping gene sequences are available for all species, some improvements are needed to make MLSA more generally applicable. The selection of an universal set of genes for all the prokaryotes, the standardization of the number and length of genes to be used or a low time consuming calculation method have been recently proposed (Glaeser and Kämpfer, 2015).

In the case of the strains of *Amycolatopsis*, it has been shown that a 315 bp variable fragment of the *gyrB* gene has a higher resolution power than the 16S rRNA gene (Everest and Meyers, 2009). This partial *gyrB* gene sequence, together with other housekeeping genes, has been proposed as a good candidate to be used for MLSA of this genus. Unfortunately, we were not able to use the *gyrB* sequence as a MLSA marker, since the sequences were partial or not present in some genomes.

### Genome comparisons

The overall similarity of the *Amycolatopsis* genomes was analyzed using several approaches.

First, we performed an overall analysis using the Genome-to-Genome Distance Calculator (GGDC) (Auch et al., 2010; Meier-Kolthoff et al., 2013, 2014), that calculates digital (*in silico*) DNA-DNA hybridization (dDDH) from the intergenomic distances and quantifies the G+C content. No G+C content differences were found among *A. mediterranei* strains. The highest G+C content differences were found between *A. halophila* DSM 45216^T^and the strains from group C (about 4% difference) (Supplementary Figure 2). In the case of strain CA-128772, the maximum and minimal G+C content differences were found with *A. balhimycina* DSM 44591^T^ (1.07%), and with *A. rifamycinica* DSM 46095^T^ (0.1%). In strain CA-126428, the maximum G+C content difference was also observed with *A. balhimycina* DSM 44591^T^ (0.62%), whereas the minimum difference (0.15%) was with *A. lexingtonensis* DSM 44653^T^. According to Meier-Kolthoff et al. (2014), within-species differences in the G+C content are almost exclusively below 1%. The observed differences below 1% between different species are probably due to the incompleteness of most of the genomes analyzed and/or inaccuracies of the species descriptions (Meier-Kolthoff et al., 2014). dDDH analysis showed that, in general, the re-association values among *Amycolatopsis* strains were in the range of 20%. However, re-association values higher than 70% were observed between some of the genomes analyzed corresponding to non-type species, indicating that they may belong to the same species (Supplementary Figure 3). This is the case of *A. orientalis* B-37 and *A. orientalis* DSM 40040^T^ with a 72.1% of re-association; *A. orientalis* HCCB10007 showed a 72.5% of re-association with *A. keratiniphila* subsp. *keratiniphila* DSM 44409^T^ and a 71.9% with *A. keratiniphila* subsp. *nogabecina* DSM 44586^T^*; A. japonica* DSM 44213^T^ showed a 72.7% of re-association with *Amycolatopsis* sp. MJM2582 and a 88.3% with *Amycolatopsis* sp. CB00013 suggesting both strains to belong to *A. japonica; Amycolatopsis* sp. M39 and *A. rubida* DSM 44637^T^ had a 93.3% of re-association; *A. keratiniphila* subsp. *nogabecina* DSM 44586^T^ and *A. keratiniphila* subsp. *keratiniphila* DSM 44409^T^ showed a 90.1%; *Amycolatopsis* sp. CB00013 had a 72.6% of re-association with *Amycolatopsis* sp. MJM2582. The rest of the dDDH obtained were below 70%, indicating that these strains belong to different species.

The re-association values between strains of group C ranged from 23 to 54%, except for the three species strains of *A. mediterranei* (U32, S699, and RB), with a 100% of re-association. In the case of the two new strains of our study, strain CA-128772 showed the maximum re-association value (45.3%) with *A. vancoresmycina* DSM 44592^T^, and strain CA-126428 had the maximum value (54.4%) with the three *A. mediterranei* strains, well below the threshold defined for strains of the same species. These results suggest that both CA-128772 and CA-126428 isolates may represent new *Amycolatopsis* strains.

Another approach used to study the similarity of the *Amycolatopsis* genomes was to perform an analysis of the Average Nucleotide Identity (ANI) and the orthology (OrthoANI) (Lee et al., 2016). The comparison of CA-128772 and CA-126428 genomes with the rest of *Amycolatopsis* genomes showed that the maximum ANI and OrthoANI values were obtained with strains included in group C. However, none of them reach the ANI threshold range (95–96%) for species delineation (Table 3). Again, as previously observed with the dDDH values, strain CA-128772 showed the highest similarity (92.0 and 91.35%) with *A. vancoresmycina* DSM 44592^T^, while strain CA-126428 showed the highest similarity (93.97 and 93.03%) with *A. mediterranei* U32. These results suggest again that CA-128772 and CA-126428 isolates may represent new *Amycolatopsis* species.

**Table 3:** OrthoANI and ANI calculations of CA-128772 and CA-126428 genomes against other *Amycolatopsis* genomes.

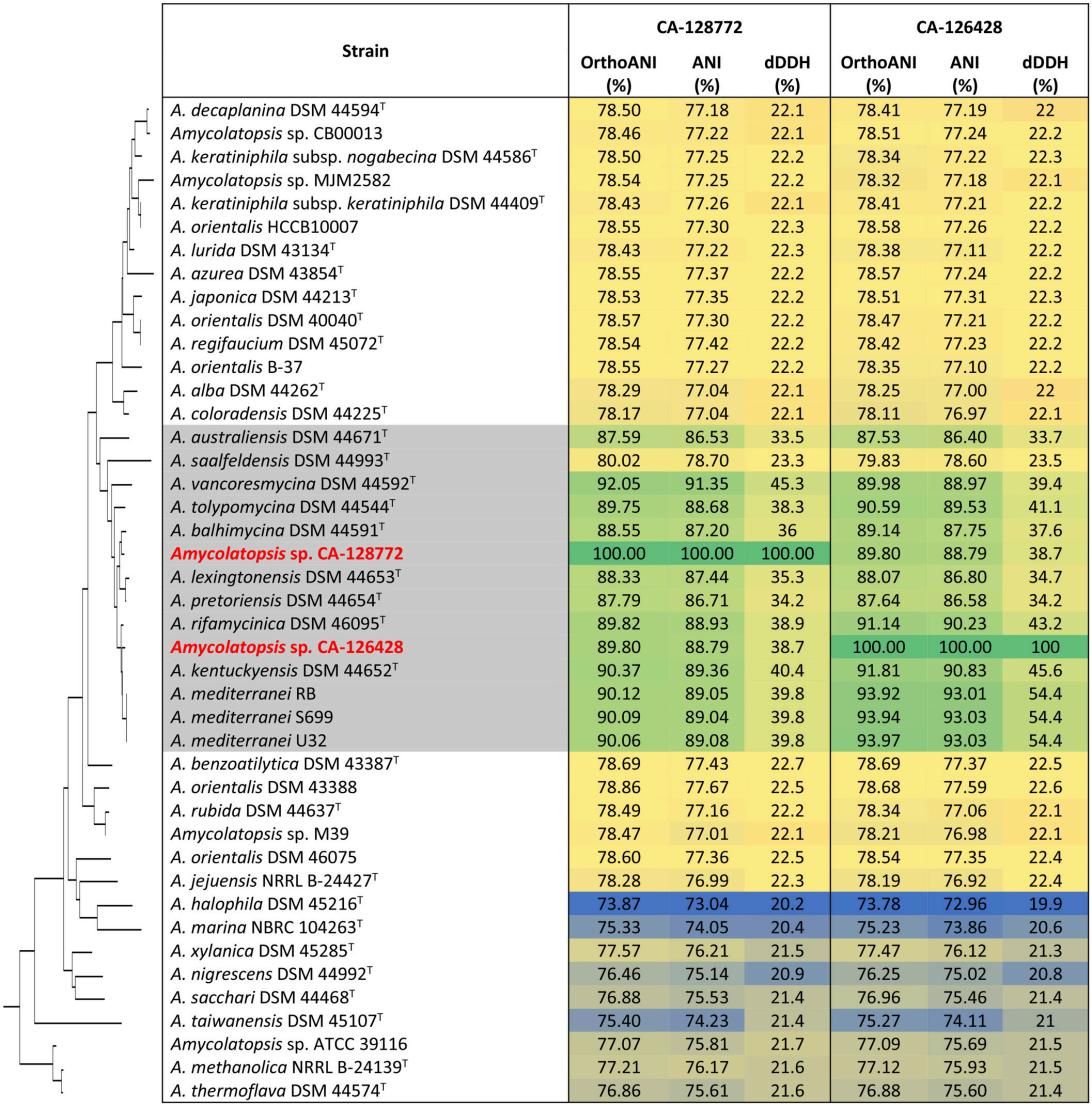

*The dDDH values have been included for comparison. Blue: lowest values, yellow: medium values, green: highest values. The strains forming a clade with CA-126428 and CA-128772 are gray-shaded. The strains are ordered in the same way as the 16S phylogenetic tree in Figure 2, which has been placed on the left for orientation*.

Several studies have confirmed that GGDC analysis yields higher correlations with classical DDH than ANI softwares (Auch et al., 2010; Meier-Kolthoff et al., 2013, 2014). Moreover, dDDH calculation is independent of genome length and is thus robust against the use of incomplete draft genomes (Auch et al., 2010).

**Figure 4:**
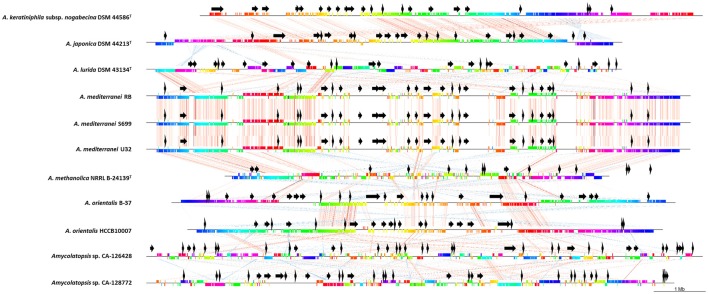
Comparative analyses of CA-126428 and CA-128772 draft genomes with 9 complete *Amycolatopsis* genomes. Horizontal straight lines represent the genomes and vertical colored bars represent different conserved genes. The vertical bars are connected by corresponding colored thin lines. Black arrows represent the position of the secondary metabolite biosynthetic pathways predicted by antiSMASH.

To compare the relative organization of the genomes, we also performed a gene synteny progressiveMauve analysis (Darling et al., 2010) of the concatenated draft genomes of strains CA-126428 and CA-128772 with nine *Amycolatopsis* complete genomes (Figure 4). The complete genomes were linearized at position 1 of their sequences. Once again, the high similarity of the three *A. mediterranei* strains was clearly observed. As previously described (Tang et al., 2016), highly conserved core regions were detected in the left and right arms of all the genomes, except for *A. lurida* DSM 43134^T^, which has a different genome rearrangement, and strains CA-126428 and CA-128772. Since the latter are draft concatenated contigs and not complete genomes, the order of the regions is altered, but homologous regions can be observed in the alignment (Figure 4). Other central regions of the chromosomes are also conserved among the genomes, especially in the *A. orientalis, A. mediterranei, A. japonica* and *A. keratiniphila* strains.

Due to the lack of resolution at the species level of the 16S rRNA gene-based phylogenies, and the difficulty to design an universal set of primers for MLSA analysis, our results derived from whole-genome content comparisons arise, as previously reported for other strains (Colston et al., 2014), as the most valuable tool to discriminate between bacterial species. As more bacterial genomes become available, the use of whole genome sequences opens new opportunities for the characterization of bacterial species.

### Primary metabolism analysis

As saprophytic bacteria, actinomycetes have a well-coordinated carbohydrate and nitrogen catabolic systems that allow the adaptation and the efficient utilization of the resources in the nutrient-limited conditions of the environment. These bacteria can produce a large diversity of extracellular enzymes to digest complex polymeric substrates and import the resulting monomers and oligomers to be used as nutrients for catabolism and anabolism and biomass generation (Genilloud, 2017b).

With the aim to analyze the global primary metabolism of strains CA-126428 and CA-128772, their genomes were functionally characterized in the KEGG PATHWAY Database (http://www.genome.jp/kegg/pathway.html) using the BlastKOALA sequence similarity tool (http://www.kegg.jp/blastkoala/). The KEGG BlastKOALA tool mapped 31.8% of the CA-126428 predicted proteins (3063 proteins) to KEGG ortholog groups, while only 19% of the CA-128772 predicted proteins (1882) could be mapped. Supplementary Table 2 presents the distribution of pathways and the number of genes annotated with BlastKOALA, and shows that the content and distribution of different metabolic pathways are very similar between both strains. Although not enough data are available because of the incompleteness of the genomes, some observations on their primary metabolism have been achieved.

A wide variety of extracellular polysaccharide-degrading enzymes have been mapped in the genomes of both *Amycolatopsis* strains including amylases, endoglucanases, xylanases, chitinases, and beta-glucosidases. However, no ligninases, agarases, mannanases, or cellulases have been detected. As in the case of many actinomycetes, some multiple transport systems to uptake specific carbohydrates are present in both genomes: such as ABC permeases and specific phosphoenolpyruvate-dependent phosphotransferase systems (PTS). In most bacteria, the PTS system plays a major role in the carbon catabolite repression (CCR), a global control system that ensures the preferential use of the different carbon sources available. CCR is mediated by the glycolytic enzyme glucose kinase, for which an ortholog has been identified in both strains. Glucose kinase converts glucose to glucose-6-phosphate, a substrate of different enzymes and pathways (glucose phosphate isomerase, glucose-6-phosphate dehydrogenase, pentose phosphate pathway, and glucose-6-phosphatase) and also plays a key role mediating CCR for enzymes involved in primary and secondary metabolism and precursors for natural product synthesis (Kwakman and Postma, 1994).

Actinomycetes carbohydrate primary metabolism is complex and characterized by the presence of multiple isoenzymes involved in single catalytic steps of glycolysis, the pentose phosphate and TCA cycle pathways. Normally, the genes encoding those enzymes do not cluster in operons and are scattered in the chromosome, subjected to different regulation depending on the carbon sources or the development stage (Genilloud, 2017b). The most important glycolytic pathway is the Embden-Meyerhof (EM) pathway (Salas et al., 1984), which is used to generate ATP and provide precursors for secondary metabolism. The EM pathway is regulated at the level of the glycolytic enzyme phosphofructokinase (Pfk) (Genilloud, 2017b), an enzyme that has not been identified so far in the genomes of strains CA-126428 and CA-128772, suggesting a different type of regulation. The enzymes involved in glucose metabolism *via* glycolysis and the pentose phosphate pathway (PPP) (Salas et al., 1984), as well as those involved in the tricarboxylic acid cycle (TCA), essential in the supply of precursors to secondary metabolism, have been mapped as expected in both isolates CA-126428 and CA-128772. The Entner-Doudoroff (ED) pathway is unusual in actinomycetes (Gunnarsson et al., 2004), although some of them contain homologs of 6-phosphogluconate dehydratase gene, as is the case of the strain CA-128772. Whether this pathway is active or not on this strain should be further investigated.

*Amycolatopsis*, as other actinomycetes, require nitrogen to ensure biomass production and to synthesize a large diversity of secondary metabolites from amino acids that are used as key precursors. Since actinomycetes are frequently isolated from nitrogen-poor environments, these bacteria have developed a complex system to efficiently retrieve nitrogen. A large diversity of extracellular proteases and peptidases are produced, as well as amino acid and oligopeptide transport systems. Some orthologous genes of these systems are also present in the genomes of strains CA-126428 and CA-128772, such as serine protease PepD, leucyl aminopeptidase, methionyl aminopeptidase, D-aminopeptidase, carboxypeptidases, amino acid, and oligopeptide transport system permeases. Different routes are followed to catabolize the families of amino acids introduced by permeases upon action of the extracellular proteases. Strains CA-126428 and CA-128772 have orthologous of most of the genes involved in these reactions, although some differences have been found between them. For example, histidine can be processed *via* formyl glutamic acid in the strain CA-128772, but in the case of CA-126428, it seems that histidine is processed *via* ergothioneine. Proline catabolism involves a proline oxidase and a pyrroline-5-carboxylate dehydrogenase to form glutamate, but the proline oxidase activity has not been mapped so far in any of the genomes. Valine dehydrogenase is responsible of the deamination of valine, leucine, and isoleucine. However, this enzyme has only been detected in strain CA-128772. Another enzyme with the same function, a branched-chain amino acid aminotransferase, is present both in CA-126428 and CA-128772. The enzymes involved in the catabolism of lysine, which usually follows the cadaverine pathway to generate glutarate, are absent in both strains. Only enzymes lysine N6-hydroxylase and lysine 2,3-aminomutase, involved in the conversion of L-lysine to N6-hydroxylysin and L-β-lysine, respectively, have been located. Enzymes involved in alanine catabolism have not been found either.

The enzymes of the shikimate pathway have been mapped in the isolates CA-126428 and CA-128772. An important number of secondary metabolites are derived from intermediates of the shikimate pathway, which is responsible of the formation of chorismate, a precursor of tryptophan, phenylalanine and tyrosine, as well as prephenate, anthranilate and p-aminobenzoate.

Interestingly, both strains possess genes involved in the degradation of aromatic compounds such as toluene, benzoate, fluorobenzoate or xylene, a trait that could be very useful for their application in bioremediation processes. So far, the genus *Amycolatopsis* has not been deeply studied in the field of bioremediation, with the exception of *Amycolatopsis tucumanensis* DSM45259^T^, which is the only species of this genus found to be resistant to copper (Albarracin et al., 2010). Another *Amycolatopsis* strain, M3-1, is able to degrade the herbicide ZJ0273, and several *Amycolatopsis* strains possess the capacity to degrade polylactic acid (PLA) (Dávila Costa and Amoroso, 2014).

In addition, we identified in both strains genes involved in antimicrobial resistance to vancomycin, beta-lactam antibiotics and cationic antimicrobial peptides. The presence of vancomycin resistance genes has been applied to the discovery of glycopeptide-producing strains using different screening approaches (Thaker et al., 2013; Truman et al., 2014), as it was the case of the ristomycin A producer *Amycolatopsis* sp. MJM2582 (Truman et al., 2014). The analysis of these genes, together with other genome mining approaches, highlights the potential to produce secondary metabolites by strains CA-128772 and CA-126428.

The former analysis of the different primary metabolism pathways present in the genomes in study supports the ability of both *Amycolatopsis* strains to utilize a broad variety of sources to ensure the supply of basic nutrients. Particularly in lichens, symbiotic partners contributions allow to colonize extreme environments and to tolerate harsh conditions. Despite the lack of information about the potential role of these strains as part of the microbial community associated to the lichen, there is also accumulating evidence that the bacterial counterparts may also contribute given their metabolic capabilities, providing carbohydrates, nitrogen sources and secondary metabolites to the microbial consortia (Scherlach and Hertweck, 2017). Future research in the physiology and primary metabolism regulation of *Amycolatopsis* spp., as well as its influence on lichen symbiosis and secondary metabolism, will benefit from the new genomic approaches and genomic-scale metabolic analyses.

### Secondary metabolite biosynthetic gene cluster analysis

Primary and secondary metabolisms are deeply interconnected in a complex network of regulatory signals that sense the environment and ensure the survival and adaptation of the microbial community (Genilloud, 2017b). The capacity to produce multiple secondary metabolites depends on the use of the available precursors and building blocks provided by the primary metabolism. In contrast to what has been observed in primary metabolism, the genes related with the production of secondary metabolites are frequently clustered, and their expression is modulated by transcriptional regulators (Genilloud, 2017b).

**Figure 5:**
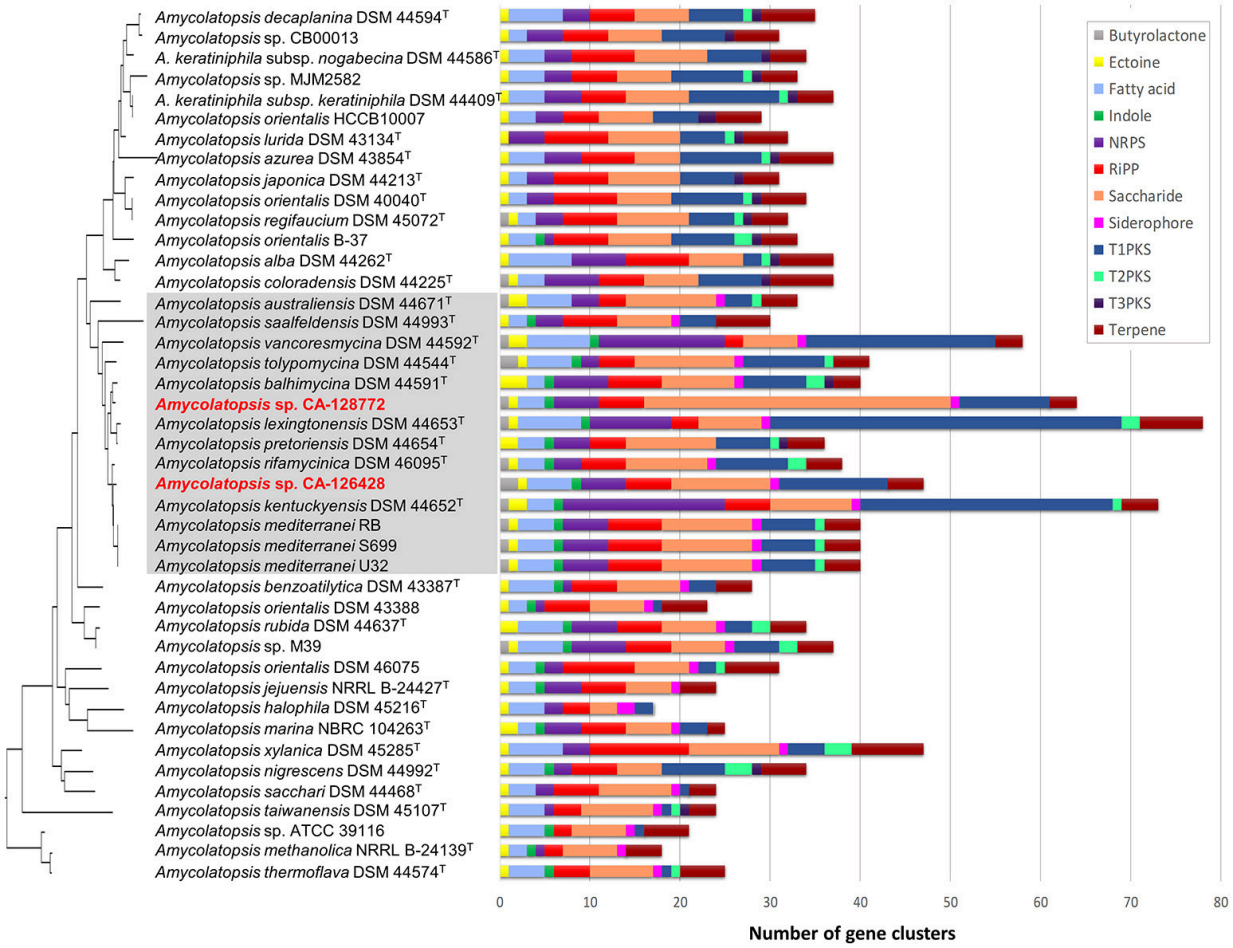
Most frequent secondary metabolite biosynthetic gene clusters predicted by antiSMASH in the genomes of *Amycolatopsis* strains. The strains are ordered in the same way as the 16S rRNA phylogenetic tree in Figure 2, which has been placed on the left axis for orientation. The strains of group C have been shaded in gray for clarification. The length of each horizontal bar corresponds with the number of BGCs, and the color code is indicated on the right.

We used the antiSMASH algorithm (Blin et al., 2017) to search for putative BGCs clusters in all the *Amycolatopsis* genomes described in Supplementary Table 1. The secondary metabolite classes examined cover all common secondary metabolites in actinomycetes (butyrolactone, ectoine, fatty acid, indole, NRPS, RiPP, saccharide, siderophore, PKS-I, PKS-II, PKS-III, and terpene) and are shown in Figure 5. As it might be expected, the number of predicted secondary metabolite biosynthetic gene clusters depends on the completeness of the genomes and the size of the contigs. Overall, the number of polyketide (PKS) and non-ribosomal peptide (NRPS) BGCs is similar among all the *Amycolatopsis* strains and correlates with the completeness of the genomes. In the case of *A. kentuckyensis* DSM 44652^T^ and *A. lexingtoniensis* DSM 44653^T^, a great number of putative PKS-I and NRPS pathways were predicted; however, these are very fragmented genomes and thus the number of predicted ORFs and BGCs increases, especially in the case of repeating, multi-modular proteins such as PKS and NRPS (Klassen and Currie, 2012).

The antiSMASH analysis of *Amycolatopsis* sequenced genomes detected many secondary metabolite BGCs when only a few metabolites have been reported to be produced by these strains, suggesting that even these well-studied species have the potential to produce new molecules (Chen et al., 2016). In the case of strains CA-126428 and CA-128772, antiSMASH predicted as many as 140 pathways for both strains given the high fragmentation level (Supplementary Table 3). In spite of the high number of predicted BGCs, only 70 clusters from CA-126428 and 55 clusters from CA-128772 show some degree of homology with known BGCs in MIBiG, and among them, only five clusters from each strain show equal or more than 50% of homology (Supplementary Table 3) suggesting the high new biosynthetic potential encoded in the genomes of the isolates CA-126428 and CA-128772.

Strain CA-128772 is richer than strain CA-126428 in saccharide pathways (Figure 5, Supplementary Table 3). A predominance of saccharide gene clusters among microbial genomes was previoulsy found in a global analysis of prokaryotic biosynthetic gene clusters (Cimermancic et al., 2014). Cell wall-associated saccharides play key roles in microbe-host and microbe-microbe interactions, while other diffusible saccharides have antibacterial activity (Cimermancic et al., 2014). The functions of many of the putative saccharide BGCs are still unknown since they are not closely related to any known gene cluster. In the case of strain CA-128772, approximately 40% of the predicted saccharide clusters do not show homology to any known cluster, in contrast to the 27% in strain CA-126428 (Supplementary Table 3).

**Figure 6:**
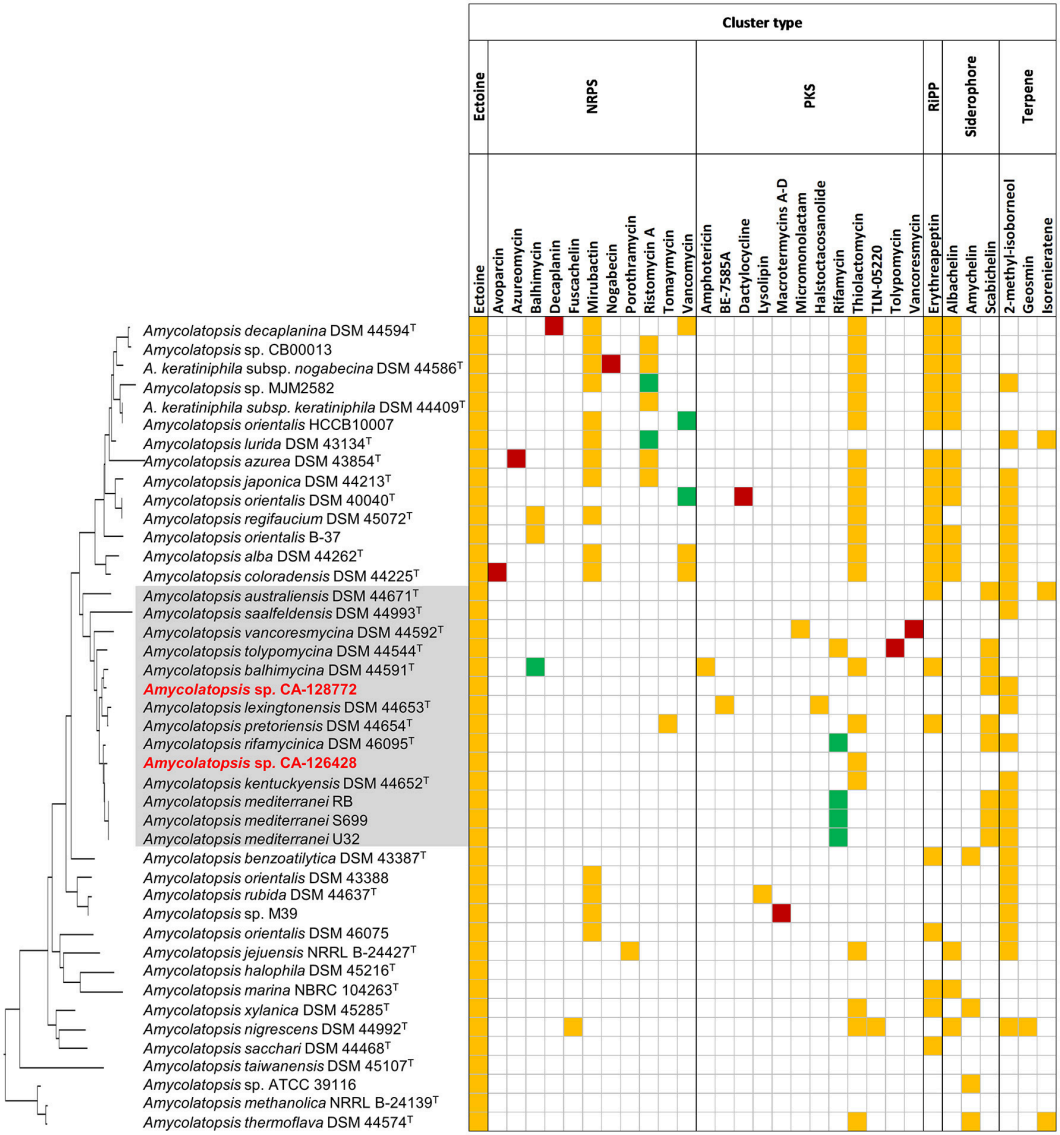
Biosynthetic gene clusters predicted in the genomes of *Amycolatopsis* with more than 70% homology with known BGCs. Yellow: predicted pathways whose encoded metabolites have not been detected in culture; green: predicted pathways whose encoded metabolites have been detected in culture; red: compounds detected in culture but not predicted by antiSMASH. The strains are ordered in the same way as the 16S rRNA phylogenetic tree in Figure 2, which has been placed on the left axis for orientation. The strains of group C have been shaded in gray for clarification.

We focused our analyses on the predicted BGC with more than 70% homology with known BGCs from the MIBiGC database (Figure 6) (Medema et al., 2015). Figure 6 shows the pathways predicted in each strain (yellow boxes), as well as the secondary metabolites that have been detected in culture fermentations (green or red boxes if the pathway has been or not predicted, respectively). Interestingly, strains CA-126428 and CA-128772 show only two and three BGCs, respectively, with more than 70% homology with known pathways (Figure 6). The rest of pathways (Figure 5, Supplementary Table 3) show low homology with known BGCs. As stated above, this fact reflects the biosynthetic potential of the strains, which may host novel BGCs encoding new secondary metabolites.

All the strains, including CA-126428 and CA-128772, were predicted to produce ectoine (1,4,5,6-tetrahydro-2-methyl-4-pyrimidinecarboxylic acid), a compatible solute with a considerable biotechnological importance that acts as stress protectant and stabilizes macromolecules against severe environmental conditions (Hamedi et al., 2013). The production of ectoine has been widely described in salt-tolerant *Streptomyces* strains (Nett et al., 2009; Zhao et al., 2016), and the antiSMASH database (https://antismash-db.secondarymetabolites.org) shows the presence of the ectoine BGC in a high number of strains belonging to the *Proteobacteria* and *Actinobacteria*. This fact suggests the capacity of adaptation of the members of this genus to different environments, since the production of ectoine may act as a protectant against desiccation or drought periods. Nevertheless, we have no further evidences about the potential role of the production of ectoine by *Amycolatopsis* in the lichen-associated microbial community.

In addition, our strains were only predicted to produce three compounds. The strain CA-128772, was predicted to produce 2-methylisoborneol, a widespread odorous-terpenoid compound (Nett et al., 2009) also shown in another 23 strains. Surprisingly, the production of this compound was not identified in any of the strains from subclades ATS and AMS. This strain was also predicted to synthetize the siderophore schabichelin (Kodani et al., 2013) as well as 9 of the 14 strains of group C (Figure 6). Strain CA-126428, as well as another 21 strains, contained a cluster encoding thiolactomycin, a thiotetronate antibiotic first described in 1982 with a broad antibacterial activity against a wide spectrum of Gram-positive and Gram-negative bacteria (Yurkovich et al., 2017). Interestingly, the production of thiolactomycin was predicted in nearly all the strains from group A except for *A. lurida* DSM 43134^T^, whereas within group C only 4 of the 14 strains may produce this compound, as well as *A. thermoflava* DSM 44574^T^, a strain from the AMS subclade (Figure 6).

An additional group of 21 strains were predicted to produce the RiPP class III lantibiotic erythreapeptin (Völler et al., 2012), from which only strains *A. australiensis* DSM 44671^T^, *A. balhimycina* DSM 44591^T^ and *A. pretoriensis* DSM 44654^T^ belonged to group C. As in the case of thiolactomycin, the production of erythreapeptin was predicted in nearly all the strains conforming group A (Figure 6), with the exception of *A. lurida* DSM 43134^T^.

Interestingly, only five strains showed a BGC with more than 70% homology with the rifamycin pathway of the ansamycin class of antibiotics, and another five strains with the vancomycin glycopeptide pathway (Chen et al., 2016). Curiously, the strains predicted to contain PKS pathways associated to rifamycin belong exclusively to group C, while strains predicted to contain a vancomycin related pathways are only present in group A.

Other biosynthetic pathways have been predicted exclusively in specific groups. These are the cases of the clusters encoding the polyketides amphotericin (Caffrey et al., 2001), BE-7585A (Sasaki et al., 2010), halstoctacosanolide (Tohyama et al., 2006), micromonolactam (Skellam et al., 2013) and the non-ribosomal peptide tomaymycin (Li et al., 2009) that were also detected only in individual strains from group C (Figure 6). The production of the glycopeptide antibiotic ristomycin A (Spohn et al., 2014) was predicted only in 7 of 14 strains from group A. Other predicted clusters are involved in the production of several siderophores such as mirubactin (Giessen et al., 2012), predicted in 11 strains from group A and in another 4 strains conforming group B, abachelin (Kodani et al., 2015), predicted in 12 strains from group A and some strains from groups D1, G, and E, and amychelin (Seyedsayamdost et al., 2011), predicted in two strains of the AMS subclade and in two strains of another AOS subgroups.

The relative position of the BGC homologous to known pathways is shown in Figure 4 (black arrows). In strains CA-126428 and CA-128772, the observed BGC distribution is not the order in the genome given that they correspond to draft concatenated and not complete genomes. Our comparison shows that strains *A. keraniphila* subsp. *nogabecina* DSM 44586^T^ and *A. japonica* DSM 44213^T^, and strains *A. mediterranei*, respectively, share similar BGCs organization, with most of the BGCs located in the non-core regions in agreement with Xu et al. (2014).

Despite the large number of BGCs detected in our strains in study, only four of them can be highly correlated to known families of compounds, with a high prevalence of BGCs showing low homology to any annotated cluster.

## Concluding remarks

The sequencing and comparative analysis of *Amycolatopsis* CA-126428 and CA-128772 genomes has contributed to improve our understanding of the taxonomic diversity and metabolic potential of the species of this genus. Both MLSA and 16S rRNA phylogenies consistently show that strains CA-126428 and CA-128772 belong to the group C of the AOS subclade. The different relative position of the strains in the 16S rRNA and MLSA phylogenies, as well as the differences observed from the genomic comparisons, suggest that strains CA-126428 and CA-128772 may represent new species, requiring further investigations to explore the uniqueness and the role of these new strains in the lichen associated microbial community.

All the *Amycolatopsis* strains analyzed have shown a large genomic potential to produce different classes of specialized metabolites restricted to few groups of species. In addition, the results of our analysis support previous reports suggesting actinomycetes as a still untapped source of novel compounds and the relevance in modern natural products drug discovery of the application of genome-based mining approaches of these species to foster the discovery of new natural products encoded by cryptic or poorly expressed BGCs.

## Author contributions

MS-H and OG designed the experiments; MS-H, IG, and CD-M performed the experiments; GM configured the bioinformatic software; and MS-H and OG wrote the manuscript.

### Conflict of interest statement

The authors declare that the research was conducted in the absence of any commercial or financial relationships that could be construed as a potential conflict of interest.
