# Supplementary material for: Comparative Genomics and Biosynthetic Potential Analysis of Two Lichen-Isolated Amycolatopsis Strains
Source: Front Microbiol. 2018 Mar 13;9:369. doi: 10.3389/fmicb.2018.00369 (PMC5859366; doi:10.3389/fmicb.2018.00369)
Supplement: Supplementary file 1 [file Image1.PDF]

frontiers

Supplementary Material

Comparative Genomics and Biosynthetic Potential Analysis of Two Lichen-Isolated *Amycolatopsis* Strains

Marina Sánchez-Hidalgo, Ignacio González, Cristian Díaz-Muñoz, Germán Martínez, Olga Genilloud*

*** Correspondence:** Olga Genilloud: olga.genilloud@medinaandalucia.es

1 **Supplementary Figure 1:** Estimates of evolutionary divergence between 16S rRNA gene sequences using the Kimura 2-parameter model (top right). Standard error estimates (accuracy of predictions) are shown below the diagonal. The strains are ordered in the same way as the phylogenetic tree in Figure 1, which has been placed upon both axes for orientation. The strains belonging to group C have been blue shaded for clarification. The heatmap legend is shown on the right. All comparisons between a genome and itself occur on the diagonal line stretching from the top left to the bottom right corners.

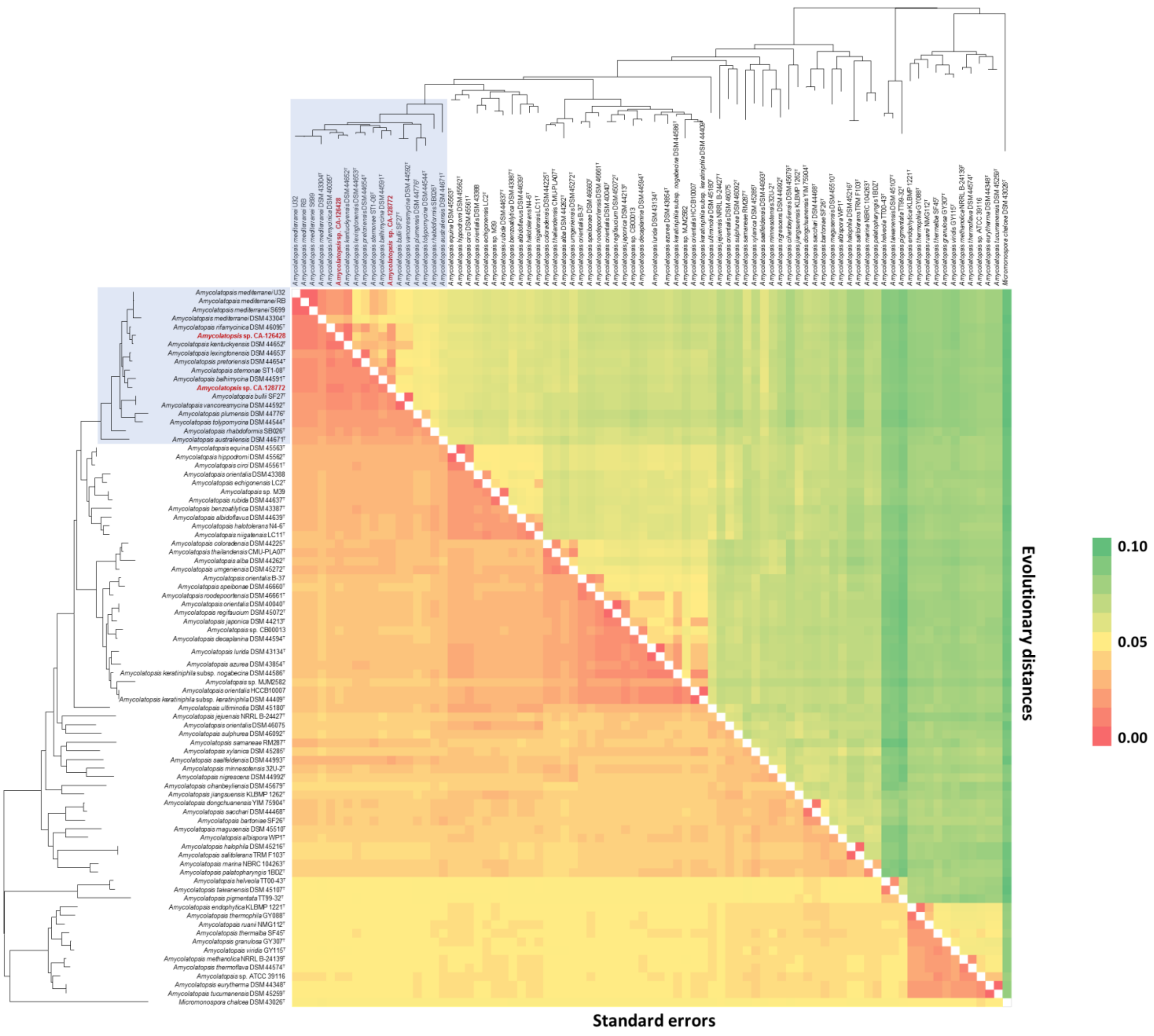
0.10
0.05
0.00
Evolutionary distances
Standard errors
