# Supplementary material for: Comparative Genomics and Biosynthetic Potential Analysis of Two Lichen-Isolated Amycolatopsis Strains
Source: Front Microbiol. 2018 Mar 13;9:369. doi: 10.3389/fmicb.2018.00369 (PMC5859366; doi:10.3389/fmicb.2018.00369)
Supplement: Supplementary file 2 [file Image2.PDF]

Supplementary Material

Comparative Genomics and Biosynthetic Potential Analysis of Two Lichen-Isolated *Amycolatopsis* Strains

Marina Sánchez-Hidalgo, Ignacio González, Cristian Díaz-Muñoz, Germán Martínez, Olga Genilloud*

*** Correspondence:** Olga Genilloud: olga.genilloud@medinaandalucia.es

1 **Supplementary Figure 2:** Percentage of G+C content differences (0-5%) calculated by GGDC. Self-genome comparisons occur on a line stretching from the top left to the bottom right corners. The strains are ordered in the same way as the 16S phylogenetic tree in Figure 2, which has been placed upon both axes for orientation. The strains belonging to group C have been shaded in blue for clarification. The heatmap legend is shown on the right.

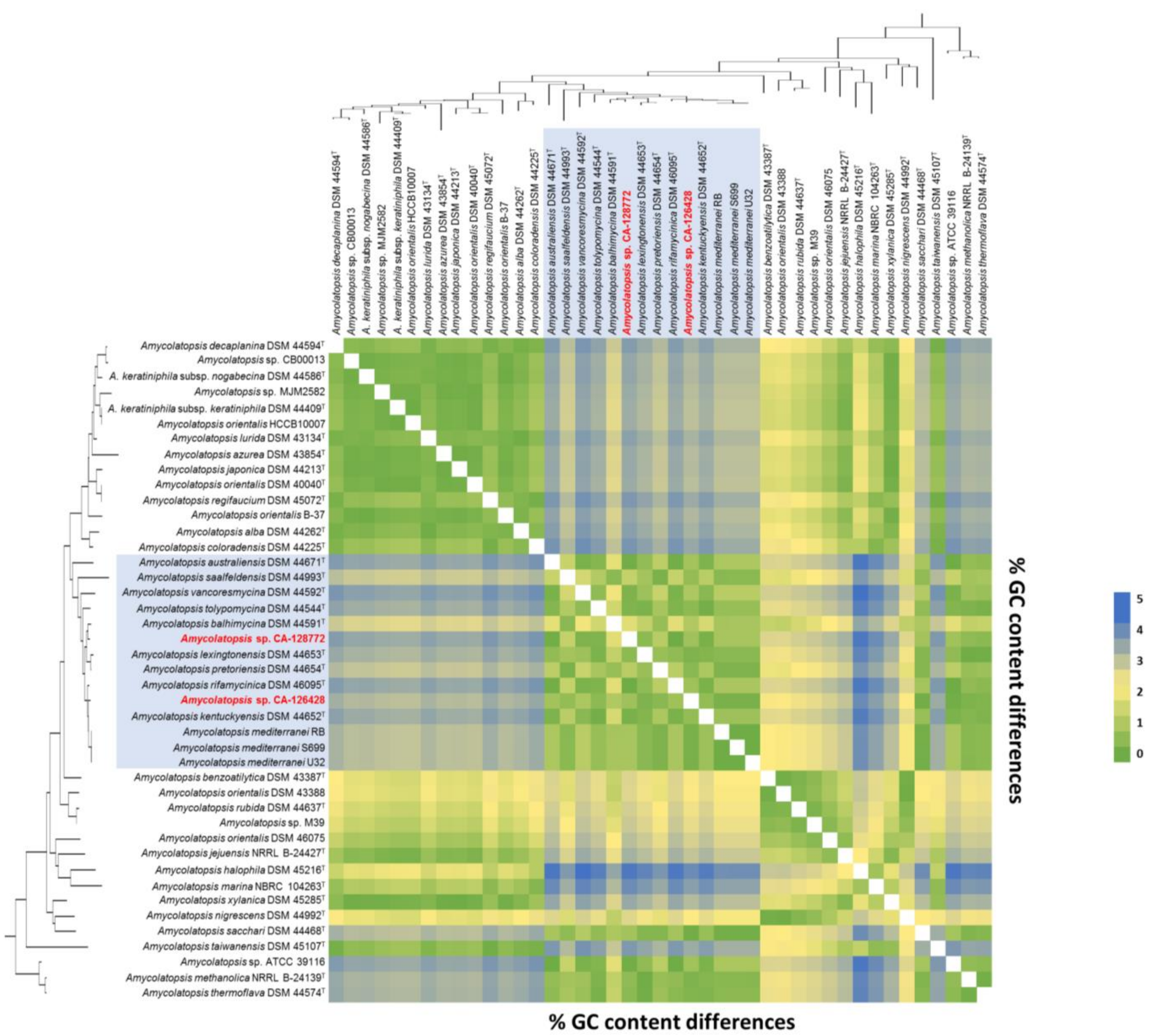
Amycolatopsis decaplanina DSM 44594T
Amycolatopsis sp. CB00013
A. keratiniphila subsp. nogabecina DSM 44586T
Amycolatopsis sp. MJM2582
A. keratiniphila subsp. keratiniphila DSM 44409T
Amycolatopsis orientalis HCCB10007
Amycolatopsis lurida DSM 43134T
Amycolatopsis azurea DSM 43854T
Amycolatopsis japonica DSM 44213T
Amycolatopsis orientalis DSM 40040T
Amycolatopsis regifaucium DSM 45072T
Amycolatopsis orientalis B-37
Amycolatopsis alba DSM 44262T
Amycolatopsis coloradensis DSM 44225T
Amycolatopsis australiensis DSM 44671T
Amycolatopsis saalfeldensis DSM 44993T
Amycolatopsis vancoresmycina DSM 44592T
Amycolatopsis tolypomycina DSM 44544T
Amycolatopsis balhimycina DSM 44591T
Amycolatopsis sp. CA-128772
Amycolatopsis lexingtonensis DSM 44653T
Amycolatopsis pretoriensis DSM 44654T
Amycolatopsis rifamycinica DSM 46095T
Amycolatopsis sp. CA-126428
Amycolatopsis kentuckyensis DSM 44652T
Amycolatopsis mediterranei RB
Amycolatopsis mediterranei S699
Amycolatopsis mediterranei U32
Amycolatopsis benzoatilytica DSM 43387T
Amycolatopsis orientalis DSM 43388
Amycolatopsis rubida DSM 44637T
Amycolatopsis sp. M39
Amycolatopsis orientalis DSM 46075
Amycolatopsis jejuensis NRRL B-24427T
Amycolatopsis halophila DSM 45216T
Amycolatopsis marina NBRC 104263T
Amycolatopsis xylanica DSM 45285T
Amycolatopsis nigrescens DSM 44992T
Amycolatopsis sacchari DSM 44468T
Amycolatopsis taiwanensis DSM 45107T
Amycolatopsis sp. ATCC 39116
Amycolatopsis methanolica NRRL B-24139T
Amycolatopsis thermoflava DSM 44574T
% GC content differences
5
4
3
2
1
0
% GC content differences
