# Supplementary material for: Comparative Genomics and Biosynthetic Potential Analysis of Two Lichen-Isolated Amycolatopsis Strains
Source: Front Microbiol. 2018 Mar 13;9:369. doi: 10.3389/fmicb.2018.00369 (PMC5859366; doi:10.3389/fmicb.2018.00369)
Supplement: Supplementary file 4 [file Table1.docx]

Supplementary Material

Comparative Genomics and Biosynthetic Potential Analysis of Two Lichen-Isolated *Amycolatopsis* Strains

**Marina Sánchez-Hidalgo, Ignacio González, Cristian Díaz-Muñoz, Germán Martínez, Olga Genilloud***

*** Correspondence:** Olga Genilloud: olga.genilloud@medinaandalucia.es

# Supplementary Table 1: *Amycolatopsis* genomes and 16S rRNA gene sequences used in this work. The NCBI sequence accession numbers are indicated.

| **Strain** | **Genome assembly reference** | **Assembly level** | **16S rRNA gene reference** | **Reference** |
| --- | --- | --- | --- | --- |
| *Amycolatopsis* sp. CA-126428 | PPHF00000000.1 | Contig | MG800320 | González et al*.,* 2005 |
| *Amycolatopsis* sp. CA-128772 | PPHG00000000.1 | Contig | MG799844 | González et al*.,* 2005 |
| *A. alba* DSM 44262^T^ | ARAF00000000.1 | Scaffold | AF051340 | Mertz and Yao, 1993 |
| *A. albidoflavus* DSM 44639^T^ | - | - | AJ252832 | Lee et al*.,* 2000 |
| *A. albispora* WP1^T^ | - | - | KT751086 | Zhang et al*.,* 2016 |
| *A. australiensis* DSM 44671^T^ | FPJG00000000.1 | Contig | AY129753 | Tan et al*.*, 2006 |
| *A. azurea* DSM 43854^T^ | MUXN00000000.1 | Scaffold | AJ400709 | Khatri et al*.*, 2014 |
| *A. balhimycina* DSM 44591^T^ | ARBH00000000.1 | Scaffold | AJ508239 | Wink et al*.,* 2003 |
| *A. bartoniae* SF26^T^ | - | - | HQ651729 | Zucchi et al*.,* 2012a |
| *A. benzoatilytica* DSM 43387^T^ | ARPK00000000.1 | Scaffold | AY957506 | Majumdar et al*.*, 2006 |
| *A. bullii* SF27^T^ | - | - | HQ651730 | Zucchi et al*.,* 2012a |
| *A. cihanbeyliensis* DSM 45679^T^ | - | - | JN989302 | Tatar et al*.,* 2013 |
| *A. circi* DSM 45561^T^ | - | - | HQ021202 | Everest et al*.,* 2011 |
| *A. coloradensis* DSM 44225^T^ | MQUQ00000000.1 | Scaffold | AJ293753 | Labeda et al*.*, 1995 |
| *A. decaplanina* DSM 44594^T^ | AOHO00000000.1 | Contig | AJ508237 | Kaur et al*.,* 2013 |
| *A. dongchuanensis* YIM 75904^T^ | - | - | JN656710 | Nie et al*.,2012* |
| *A. echigonensis* LC2^T^ | - | - | AB248535 | Ding et al*.,* 2007 |
| *A. endophytica* KLBMP 1221^T^ | - | - | HM153799 | Miao et al*.,* 2011 |
| *A. equina* DSM 45563^T^ | - | - | HQ021204 | Everest et al*.,* 2011 |
| *A. eurytherma* DSM 44348^T^ | - | - | AJ000285 | Kim et al*.,* 2002 |
| *A. granulosa* GY307^T^ | - | - | AF466101 | Zucchi et al., 2012b |
| *A. halophila* DSM 45216^T^ | AZAK00000000.1 | Scaffold | FJ606836 | Tang et al*.,* 2010 |
| *A. halotolerans* N4-6^T^ | - | - | DQ000196 | Lee, 2006 |
| *A. helveola* TT00-43^T^ | - | - | AB327253 | Tamura et al*.,* 2010 |
| *A. hippodromi* DSM 45562^T^ | - | - | HQ021203 | Everest et al*.,* 2011 |
| *A. hippodromi* S3.6^T^ | - | - | HQ021203 | Everest et al*.,* 2011 |
| *A. japonica* DSM 44213^T^ | CP008953.1 | Complete | AJ508236 | Stegmann et al*.,* 2014 |
| *A. jejuensis* NRRL B-24427^T^ | JNYZ00000000.1 | Contig | DQ000200 | Lee, 2006 |
| *A. jiangsuensis* KLBMP 1262^T^ | - | - | JQ819253 | Xing et al*.,* 2013 |
| *A. kentuckyensis* DSM 44652^T^ | MUMI00000000.1 | Contig | AY183357 | Labeda et al*.*, 2003 |
| *A. keratiniphila* subsp. *keratiniphila* DSM 44409^T^ | LQMT00000000.2 | Contig | AJ278496 | Wink et al*.,* 2003 |
| *A. keratiniphila* subsp. *nogabecina* DSM 44586^T^ | LT629789.1 | Complete | AJ508238 | Wink et al*.,* 2003 |
| *A. lexingtonensis* DSM 44653^T^ | MUMJ00000000.1 | Contig | AY183358 | Labeda et al*.*, 2003 |
| *A. lurida* DSM 43134^T^ | CP007219.1 | Complete | AJ293755 | Kwun and Hong, 2014 |
| *A. magusensis* DSM 45510^T^ | - | - | HQ157190 | Camas et al*.,* 2013 |
| *A. marina* NBRC 104263^T^ | FOKG00000000.1 | Scaffold | EU329845 | Bian et al*.*, 2009 |
| *A. mediterranei* DSM 43304^T^ | - | - | AJ293754 | Lechevalier et al*.,* 1986 |
| *A. mediterranei* RB | CP003777.1 | Complete | CP003777.1 | Zhao et al., 2010 |
| *A. mediterranei* S699 | CP003729.1 | Complete | CP003729.1 | Tang et al., 2012 |
| *A. mediterranei* U32 | CP002000.1 | Complete | CP002000.1 | Zhao et al*.*, 2010 |
| *A. methanolica* NRRL B-24139^T^ | CP009110.1 | Complete | AJ249135 | Tang et al, 2016 |
| *A. minnesotensis* 32U-2^T^ | - | - | DQ076482 | Lee et al*.,* 2006 |
| *A. nigrescens* DSM 44992^T^ | ARVW00000000.1 | Contig | DQ486888 | Groth et al., 2007 |
| *A. niigatensis* LC11^T^ | - | - | AB248537 | Ding et al*.,* 2007 |
| *A. orientalis* B-37 | CP016174.1 | Complete | CP016174.1 | Lei et al*.*, 2015 |
| *A. orientalis* DSM 40040^T^ | ASJB00000000.1 | Contig | AJ400711 | Lechevalier et al*.,* 1986 |
| *A. orientalis* DSM 43388 | ASXG00000000.1 | Contig | ASXG00000000.1 | Jeong et al*.,* 2013 |
| *A. orientalis* DSM 46075 | ASXH00000000.1 | Contig | ASXH00000000.1 | Jeong et al*.,* 2013 |
| *A. orientalis* HCCB10007 | CP003410.1 | Complete | CP003410.1 | Xu et al*.*, 2014 |
| *A. palatopharyngis* 1BDZ^T^ | - | - | AF479268 | Huang et al*.,* 2004 |
| *A. pigmentata* TT99-32^T^ | - | - | AB327254 | Tamura et al*.,* 2010 |
| *A. plumensis* DSM 44776^T^ | - | - | AY262825 | Saintpierre-Bonaccio et al*.,* 2005 |
| *A. pretoriensis* DSM 44654^T^ | MUMK00000000.1 | Scaffold | AY183356 | Labeda et al*.*, 2003 |
| *A. regifaucium* DSM 45072^T^ | LQCI00000000.1 | Contig | AY129760 | Tan et al*.*, 2007 |
| *A. rhabdoformis* SB026^T^ | - | - | KF779477 | Souza et al*.,* 2015 |
| *A. rifamycinica* DSM 46095^T^ | JMQI00000000.1 | Contig | AY083603 | Saxena et al*.,* 2014 |
| *A. roodepoortensis* DSM 46661^T^ | - | - | KF771262 | Everest et al*.,* 2014 |
| *A. ruanii* NMG112^T^ | - | - | HQ668524 | Zucchi et al., 2012b |
| *A. rubida* DSM 44637^T^ | FOWC00000000.1 | Contig | AF222022 | Huang et al*.,* 2001 |
| *A. saalfeldensis* DSM 44993^T^ | FOEF00000000.1 | Scaffold | DQ792500 | Carlsohn et al*.,* 2007 |
| *A. sacchari* DSM 44468^T^ | FORP00000000.1 | Contig | AF223354 | Goodfellow et al*.,* 2001 |
| *A. salitolerans* TRM F103^T^ | - | - | HQ436534 | Guan et al*.,* 2012 |
| *A. samaneae* RM287^T^ | - | - | GQ381310 | Duangmal et al*.,* 2011 |
| *Amycolatopsis* sp. ATCC 39116 | AFWY00000000.3 | Contig | AM263202 | Davis et al*.,* 2012 |
| *Amycolatopsis* sp. CB00013 | LIWC00000000.1 | Scaffold | KT722894 | Yan et al, 2016 |
| *Amycolatopsis* sp. M39 | [LWSF00000000.1](https://www.ncbi.nlm.nih.gov/nuccore/LWSF00000000.1) | Scaffold | KX161771 | Beemelmanns et al*.,* 2017 |
| *Amycolatopsis* sp. MJM2582 | JPLW00000000.1 | Scaffold | KP872909 | Kwun et al*.*, 2014 |
| *A. speibonae* DSM 4660^T^ | - | - | KF771257 | Everest et al*.,* 2014 |
| *A. stemonae* ST1-08^T^ | - | - | LC011703 | Klykleung et al*.,* 2015 |
| *A. sulphurea* DSM 46092^T^ | - | - | AJ293756 | Lechevalier et al., 1986 |
| *A. taiwanensis* DSM 45107^T^ | JAFB00000000.1 | Scaffold | DQ160215 | Tseng et al*.,* 2006 |
| *A. thailandensis* CMU-PLA07^T^ | - | - | FJ581021 | Chomchoei et al*.,* 2011 |
| *A. thermalba* SF45^T^ | - | - | HQ668525 | Zucchi et al., 2012b |
| *A. thermoflava* DSM 44574^T^ | AXBH00000000.1 | Scaffold | AF052390 | Chun et al*.*, 1999 |
| *A. thermophila* GY088^T^ | - | - | AY129774 | Zucchi et al., 2012c |
| *A. tolypomycina* DSM 44544^T^ | FNSO00000000.1 | Contig | AJ293757 | Wink et al*.,* 2003 |
| *A. tucumanensis* DSM 45259^T^ | - | - | DQ886938 | Albarracín et al*.,* 2010 |
| *A. ultiminotia* DSM 45180^T^ | - | - | FM177516 | Lee, 2009 |
| *A. umgeniensis* DSM 45272^T^ | - | - | DQ110876 | Everest et al*.,* 2013 |
| *A. vancoresmycina* DSM 44592^T^ | JNYY00000000.1 | Contig | AJ508240 | Kaur et al*.*, 2014 |
| *A. viridis* GY115^T^ | - | - | AF466095 | Zucchi et al., 2012c |
| *A. xylanica* DSM 45285^T^ | FNON00000000.1 | Scaffold | FJ529702 | Chen et al*.,* 2010 |
| *Micromonospora chalcea* DSM 43026^T^ |  |  | X92594 | Foulerton, 1905 |

**References**

Bian, J., Li, Y., Wang, J., Song, F.H., Liu, M., Dai, H.Q., et al. (2009*). Amycolatopsis marina* sp. nov., an actinomycete isolated from an ocean sediment*. Int. J. Syst. Evol. Microbiol*. 59, 477–481. doi:10.1099/ijs.0.000026-0

Camas, M., Sahin, N., Sazak, A., Sproer, C., and Klenk, H.P. (2013). *Amycolatopsis magusensis* sp. nov., isolated from soil. *Int. J. Syst. Evol. Microbiol*. 63, 1254–1260. doi:10.1099/ijs.0.042770-0

Carlsohn, M. R., Groth, I., Tan, G. Y. A., Schutze, B., Saluz, H. P., Munder, T., et al. (2007). *Amycolatopsis saalfeldensis* sp. nov., a novel actinomycete isolated from a medieval alum slate mine. *Int. J. Syst. Evol. Microbiol*. 57, 1640–1646. doi:10.1099/ijs.0.64903-0

Chen, J., Su, J.J., Wei, Y.Z., Li, Q.P., Yu, L. Y., Liu, H. Y., et al. (2010). *Amycolatopsis xylanica* sp. nov., isolated from soil. *Int. J. Syst. Evol. Microbiol.* 60, 2124–2128. doi:10.1099/ijs.0.016865-0

Chomchoei, A., Pathom-Aree, W., Yokota, A., Kanongnuch, C., and Lumyong, S. (2011). *Amycolatopsis thailandensis* sp. nov., a poly(L-lactic acid)-degrading actinomycete, isolated from soil. *Int. J. Syst. Evol. Microbiol.* 61, 839–843. doi:10.1099/ijs.0.023564-0

Chun, J., Kim, S. B., Oh, Y. K., Seong, C.-N., Lee, D.-H., Bae, K. S., et al. (1999). *Amycolatopsis thermoflava* sp. nov., a novel soil actinomycete from Hainan Island, China. *Int. J. Syst. Bacteriol.* 49, 1369–1373. doi:10.1099/00207713-49-4-1369

Davis, J. R., Goodwin, L. A., Woyke, T., Teshima, H., Bruce, D., Detter, C., et al. (2012). Genome sequence of *Amycolatopsis* sp. strain ATCC 39116, a plant biomass-degrading actinomycete. *J. Bacteriol*. 194, 2396–2397. doi:10.1128/JB.00186-12

Ding, L., Hirose, T., and Yokota, A. (2007). *Amycolatopsis echigonensis* sp. nov. and *Amycolatopsis niigatensis* sp. nov., novel actinomycetes isolated from a filtration substrate. *Int. J. Syst. Evol. Microbiol*. 57, 1747–1751. doi:10.1099/ijs.0.64791-0

Duangmal, K., Mingma, R., Pathom-aree, W., Thamchaipenet, A., Inahashi, Y., Matsumoto, A., et al. (2011). *Amycolatopsis samaneae* sp. nov., isolated from roots of *Samanea saman* (Jacq.) Merr. *Int. J. Syst. Evol. Microbiol*. 61, 951–955. doi:10.1099/ijs.0.022699-0

Everest, G. J., le Roes-Hill, M., Omorogie, C., Cheung, S.-K., Cook, A. E., Goodwin, C. M., et al. (2013). *Amycolatopsis umgeniensis* sp. nov., isolated from soil from the banks of the Umgeni River in South Africa. *Antonie Van Leeuwenhoek* 103, 673–681. doi:10.1007/s10482-012-9851-7

Everest, G. J., le Roes-Hill, M., Rohland, J., Enslin, S., and Meyers, P. R. (2014). *Amycolatopsis roodepoortensis* sp. nov. and *Amycolatopsis speibonae* sp. nov.: antibiotic-producing actinobacteria isolated from South African soils. *J. Antibiot. (Tokyo).* 67, 813–818. doi:10.1038/ja.2014.79

Goodfellow, M., Kim, S. B., Minnikin, D. E., Whitehead, D., Zhou, Z. H., and Mattinson-Rose, A. D. (2001). *Amycolatopsis sacchari* sp. nov., a moderately thermophilic actinomycete isolated from vegetable matter. *Int. J. Syst. Evol. Microbiol*. 51, 187–193. doi:10.1099/00207713-51-1-187

Groth, I., Tan, G. Y. A., Gonzalez, J. M., Laiz, L., Carlsohn, M. R., Schutze, B., et al. (2007). *Amycolatopsis nigrescens* sp. nov., an actinomycete isolated from a Roman catacomb. Int. *J. Syst. Evol. Microbiol*. 57, 513–519. doi:10.1099/ijs.0.64602-0

Guan, T. W., Xia, Z.-F., Tang, S.-K., Wu, N., Chen, Z.-J., Huang, Y., et al. (2012). *Amycolatopsis salitolerans* sp. nov., a filamentous actinomycete isolated from a hypersaline habitat. *Int. J. Syst. Evol. Microbiol.* 62, 23-27.doi:10.1099/ijs.0.030031-0

Huang, Y., Paściak, M., Liu, Z., Xie, Q., and Gamian, A. (2004). *Amycolatopsis palatopharyngis* sp. nov., a potentially pathogenic actinomycete isolated from a human clinical source. *Int. J. Syst. Evol. Microbiol*. 54, 359–363. doi:10.1099/ijs.0.02685-0

Huang, Y., Qi, W., Lu, Z., Liu, Z., and Goodfellow, M. (2001). *Amycolatopsis rubida* sp. nov., a new *Amycolatopsis* species from soil. *Int. J. Syst. Evol. Microbiol.* 51, 1093–1097. doi:10.1099/00207713-51-3-1093

Jeong, H., Sim, Y. M., Kim, H. J., Lee, Y. J., Lee, D.-W., Lim, S.-K., et al. (2013). Genome sequences of *Amycolatopsis orientalis* subsp. *orientalis* strains DSM 43388 and DSM 46075. *Genome Announc*. 1, e00545-13-e00545-13. doi:10.1128/genomeA.00545-13

Kaur, N., Kumar, S., Bala, M., Raghava, G. P. S., and Mayilraj, S. (2013). Draft genome sequence of *Amycolatopsis decaplanina* strain DSM 44594T. *Genome Announc*. 1, e00138-13-e00138-13. doi:10.1128/genomeA.00138-13

Kaur, N., Kumar, S., and Mayilraj, S. (2014). Genome sequencing and annotation of *Amycolatopsis vancoresmycina* strain DSM 44592T. *Genomics Data* 2, 16–17. doi:10.1016/j.gdata.2013.10.006

Khatri, I., Subramanian, S., and Mayilraj, S. (2014). Genome sequencing and annotation of *Amycolatopsis azurea* DSM 43854T. *Genomics Data* 2, 44–45. doi:10.1016/j.gdata.2013.12.003

Kim, B., Sahin, N., Tan, G. Y. A., Zakrzewska-Czerwinska, J., and Goodfellow, M. (2002). *Amycolatopsis eurytherma* sp. nov., a thermophilic actinomycete isolated from soil. *Int. J. Syst. Evol. Microbiol.* 52, 889–894. doi:10.1099/ijs.0.02060-0

Klykleung, N., Tanasupawat, S., Pittayakhajonwut, P., Ohkuma, M., and Kudo, T. (2015). *Amycolatopsis stemonae* sp. nov., isolated from a thai medicinal plant. *Int. J. Syst. Evol. Microbiol.* 65, 3894–3899. doi:10.1099/ijsem.0.000509.

Kwun, M. J., Cheng, J., Yang, S. H., Lee, D. R., Suh, J. W., and Hong, H. J. (2014a). Draft genome sequence of ristocetin-producing strain *Amycolatopsis* sp. strain MJM2582 isolated in South Korea. *Genome Announc.* 2, e01091-14. doi:10.1128/genomeA.01091-14.

Kwun, M. J., and Hong, H. J. (2014b). Draft genome sequence of *Amycolatopsis lurida* NRRL 2430, producer of the glycopeptide family antibiotic ristocetin. *Genome Announc*. 2, e01050-14-e01050-14. doi:10.1128/genomeA.01050-14.

Labeda, D. P. (1995). *Amycolatopsis coloradensis* sp. nov., the avoparcin (LL-AV290)-producing strain. *Int. J. Syst. Bacteriol*. 45, 124–127. doi:10.1099/00207713-45-1-124.

Labeda, D. P. (2003). *Amycolatopsis kentuckyensis* sp. nov., *Amycolatopsis lexingtonensis* sp. nov. and *Amycolatopsis pretoriensis* sp. nov., isolated from equine placentas. *Int. J. Syst. Evol. Microbiol.* 53, 1601–1605. doi:10.1099/ijs.0.02691-0.

Lee, S. D. (2006)*. Amycolatopsis jejuensis* sp. nov. and *Amycolatopsis halotolerans* sp. nov., novel actinomycetes isolated from a natural cave. *Int. J. Syst. Evol. Microbiol*. 56, 549–553. doi:10.1099/ijs.0.63881-0.

Lee, S. D. (2009). *Amycolatopsis ultiminotia* sp. nov., isolated from rhizosphere soil, and emended description of the genus *Amycolatopsis*. *Int. J. Syst. Evol. Microbiol*. 59, 1401–1404. doi:10.1099/ijs.0.006577-0.

Lee, S. D., Kim, E. S., and Hah, Y. C. (2000). Phylogenetic analysis of the genera *Pseudonocardia* and *Actinobispora* based on 16S ribosomal DNA sequences. *FEMS Microbiol. Lett.* 182, 125–129. doi:10.1111/j.1574-6968.2000.tb08885.x.

Lee, S. D., Kinkel, L. L., and Samac, D. A. (2006). *Amycolatopsis minnesotensis* sp. nov., isolated from a prairie soil. *Int. J. Syst. Evol. Microbiol*. 56, 265–269. doi:10.1099/ijs.0.63907-0.

Lei, X., Yuan, F., Shi, Y., Li, X., Wang, L., and Hong, B. (2015). Draft genome sequence of norvancomycin-producing strain *Amycolatopsis orientalis* CPCC200066. *Genome Announc.* 3, e00296-15. doi:10.1128/genomeA.00296-15.

Majumdar, S., Prabhagaran, S., Shivaji, S., and Lal, R. (2006). Reclassification of *Amycolatopsis orientalis* DSM 43387 as *Amycolatopsis benzoatilytica* sp. nov. *Int. J. Syst. Evol. Microbiol.* 56, 199–204. doi:10.1099/ijs.0.63766-0.

Mertz, F. P., and Yao, R. C. (1993). *Amycolatopsis alba* sp. nov., isolated from soil. *Int. J. Syst. Bacteriol.* 43, 715–720. doi:10.1099/00207713-43-4-715.

Miao, Q., Qin, S., Bian, G. K., Yuan, B., Xing, K., Zhang, Y. J., et al. (2011). *Amycolatopsis endophytica* sp. nov., a novel endophytic actinomycete isolated from oil-seed plant *Jatropha curcas* L. *Antonie Van Leeuwenhoek*. 100, 333–339. doi:10.1007/s10482-011-9588-8.

Nie, G.-X., Ming, H., Li, S., Zhou, E.-M., Cheng, J., Tang, X., et al. (2012). *Amycolatopsis dongchuanensis* sp. nov., an actinobacterium isolated from soil. *Int. J. Syst. Evol. Microbiol.* 62, 2650–2656. doi:10.1099/ijs.0.038125-0.

Saintpierre-Bonaccio, D., Amir, H., Pineau, R., Tan, G. Y. A., and Goodfellow, M. (2005). *Amycolatopsis plumensis* sp. nov., a novel bioactive actinomycete isolated from a New-Caledonian brown hypermagnesian ultramafic soil. *Int. J. Syst. Evol. Microbiol*. 55, 2057–2061. doi:10.1099/ijs.0.63630-0.

Saxena, A., Kumari, R., Mukherjee, U., Singh, P., and Lal, R. (2014). Draft genome sequence of the rifamycin producer *Amycolatopsis rifamycinica* DSM 46095. *Genome Announc.* 2, e00662-14-e00662-14. doi:10.1128/genomeA.00662-14.

Souza, W. R., Silva, R. E., Goodfellow, M., Busarakam, K., Figueiro, F. S., Ferreira, D., et al. (2015). *Amycolatopsis rhabdoformis* sp. nov., an actinomycete isolated from a tropical forest soil. *Int. J. Syst. Evol. Microbiol*. 65, 1786–1793. doi:10.1099/ijs.0.000176.

Stegmann, E., Albersmeier, A., Spohn, M., Gert, H., Weber, T., Wohlleben, W., et al. (2014). Complete genome sequence of the actinobacterium *Amycolatopsis japonica* MG417-CF17T (=DSM 44213T) producing (S,S)-N,N′-ethylenediaminedisuccinic acid. *J. Biotechnol.* 189, 46–47. doi:10.1016/j.jbiotec.2014.08.034.

Tamura, T., Ishida, Y., Otoguro, M., and Suzuki, K. (2010). *Amycolatopsis helveola* sp. nov. and *Amycolatopsis pigmentata* sp. nov., isolated from soil. *Int. J. Syst. Evol. Microbiol*. 60, 2629–2633. doi:10.1099/ijs.0.017871-0.

Tan G, Robinson S, Lacey E, G. M. (2006). *Amycolatopsis australiensis* sp. nov., an actinomycete isolated from arid soils. *Int. J. Syst. Evol. Microbiol*. 56, 2297–2301. doi:10.1099/ijs.0.64260-0.

Tan, G. Y. A., Robinson, S., Lacey, E., Brown, R., Kim, W., and Goodfellow, M. (2007). *Amycolatopsis regifaucium* sp. nov., a novel actinomycete that produces kigamicins*. Int. J. Syst. Evol. Microbiol.* 57, 2562–2567. doi:10.1099/ijs.0.64974-0.

Tang, B., Zhao, W., Zheng, H., Zhuo, Y., Zhang, L., and Zhao, G.-P. (2012). Complete Genome Sequence of *Amycolatopsis mediterranei* S699 Based on *De Novo* Assembly *via* a Combinatorial Sequencing Strategy. *J. Bacteriol*. 194, 5699–5700. doi:10.1128/JB.01295-12.

Tang, S. K., Wang, Y., Guan, T. W., Lee, J. C., Kim, C. J., and Li, W. J. (2010). *Amycolatopsis halophila* sp. nov., a halophilic actinomycete isolated from a salt lake. *Int. J. Syst. Evol. Microbiol.* 60, 1073–1078. doi:10.1099/ijs.0.012427-0.

Tatar, D., Sazak, A., Guven, K., Cetin, D., and Sahin, N. (2013). *Amycolatopsis cihanbeyliensis* sp. nov., a halotolerant actinomycete isolated from a salt mine. *Int. J. Syst. Evol. Microbiol.* 63, 3739–3743. doi:10.1099/ijs.0.050963-0.

Tseng M, Yang S, Li W, J. C. (2006). *Amycolatopsis taiwanensis* sp. nov., from soil. *Int. J. Syst. Evol. Microbiol.* 56, 1811–1815. doi:10.1099/ijs.0.64149-0.

Wink, J. M., Kroppenstedt, R. M., Ganguli, B. N., Nadkarni, S. R., Schumann, P., Seibert, G., et al. (2003). Three new antibiotic producing species of the genus *Amycolatopsis, Amycolatopsis balhimycina* sp. nov., *A. tolypomycina* sp. nov., *A. vancoresmycina* sp. nov., and description of *Amycolatopsis keratiniphila* subsp. *keratiniphila* subsp. nov. and *A. keratiniphila* subsp. *nogabecina* subsp. nov. *Syst. Appl. Microbiol*. 26, 38–46. doi:10.1078/072320203322337290.

Xing, K., Liu, W., Zhang, Y.-J., Bian, G.-K., Zhang, W.-D., Tamura, T., et al. (2013). *Amycolatopsis jiangsuensis* sp. nov., a novel endophytic actinomycete isolated from a coastal plant in Jiangsu, China. *Antonie Van Leeuwenhoek* 103, 433–439. doi:10.1007/s10482-012-9823-y.

Yan, X., Ge, H., Huang, T., Hindra, Yang, D., Teng, Q., et al. (2016). Strain prioritization and genome mining for enediyne natural products. *MBio* 7, e02104-16. doi:10.1128/mBio.02104-16.

Zhang, G., Wang, L., Li, J., and Zhou, Y. (2016). *Amycolatopsis albispora* sp. nov., isolated from deep-sea sediment. *Int. J. Syst. Evol. Microbiol*. 66, 3860–3864. doi:10.1099/ijsem.0.001277.

Zhao, W., Zhong, Y., Yuan, H., Wang, J., Zheng, H., Wang, Y., et al. (2010). Complete genome sequence of the rifamycin SV-producing *Amycolatopsis mediterranei* U32 revealed its genetic characteristics in phylogeny and metabolism. *Cell Res*. 20, 1096–1108. doi:10.1038/cr.2010.87.

Zucchi, T. D., Bonda, A. N. V., Frank, S., Kim, B.-Y., Kshetrimayum, J. D., and Goodfellow, M. (2012a). *Amycolatopsis bartoniae* sp. nov. and *Amycolatopsis bullii* sp. nov., mesophilic actinomycetes isolated from arid Australian soils*. Antonie Van Leeuwenhoek* 102, 91–98. doi:10.1007/s10482-012-9716-0.

Zucchi, T. D., Tan, G. Y. A., Bonda, A. N. V., Frank, S., Kshetrimayum, J. D., and Goodfellow, M. (2012b). *Amycolatopsis granulosa* sp. nov., *Amycolatopsis ruanii* sp. nov. and *Amycolatopsis thermalba* sp. nov., thermophilic actinomycetes isolated from arid soils. Int. J. Syst. Evol. Microbiol. 62, 1245–1251. doi:10.1099/ijs.0.031039-0.

Zucchi, T. D., Tan, G. Y. A., and Goodfellow, M. (2012c). *Amycolatopsis thermophila* sp. nov. and *Amycolatopsis viridis* sp. nov., thermophilic actinomycetes isolated from arid soil. *Int. J. Syst. Evol. Microbiol*. 62, 168–172. doi:10.1099/ijs.0.029256-0.
