# Supplementary material for: Comparative Genomics and Biosynthetic Potential Analysis of Two Lichen-Isolated Amycolatopsis Strains
Source: Front Microbiol. 2018 Mar 13;9:369. doi: 10.3389/fmicb.2018.00369 (PMC5859366; doi:10.3389/fmicb.2018.00369)
Supplement: Supplementary file 5 [file Table2.DOCX]

Supplementary Material

Comparative Genomics and Biosynthetic Potential Analysis of Two Lichen-Isolated *Amycolatopsis* Strains

**Marina Sánchez-Hidalgo, Ignacio González, Cristian Díaz-Muñoz, Germán Martínez, Olga Genilloud***

*** Correspondence:** Olga Genilloud: olga.genilloud@medinaandalucia.es

# Supplementary Table 2: Metabolic pathways and number of genes annotated with BlastKOALA in the genomes of strains CA-126428 and CA-128772. Global and overview pathways are not shown in the table.

| **Pathways** | | **Number of genes** | |
| --- | --- | --- | --- |
|  |  | **CA-126428** | **CA-128772** |
| **Metabolism** | |  |  |
|  | **Carbohydrate metabolism** | **329** | **294** |
|  | Glycolysis / Gluconeogenesis | 28 | 25 |
|  | Citrate cycle (TCA cycle) | 22 | 19 |
|  | Pentose phosphate pathway | 19 | 15 |
|  | Pentose and glucuronate interconversions | 14 | 12 |
|  | Fructose and mannose metabolism | 25 | 24 |
|  | Galactose metabolism | 15 | 15 |
|  | Ascorbate and aldarate metabolism | 7 | 6 |
|  | Starch and sucrose metabolism | 27 | 24 |
|  | Amino sugar and nucleotide sugar metabolism | 38 | 35 |
|  | Piruvate metabolism | 26 | 24 |
|  | Glyoxylate and dicarboxylate metabolism | 34 | 30 |
|  | Propanoate metabolism | 26 | 27 |
|  | Butanoate metabolism | 26 | 22 |
|  | C5-Branched dibasic acid metabolism | 11 | 6 |
|  | Inositol phosphate metabolism | 11 | 10 |
|  | **Energy metabolism** | **115** | **84** |
|  | Oxidative phosphorylation | 39 | 25 |
|  | Carbon fixation pathways in prokaryotes | 25 | 22 |
|  | Methane metabolism | 20 | 14 |
|  | Nitrogen metabolism | 13 | 10 |
|  | Sulfur metabolism | 18 | 13 |
|  | **Lipid metabolism** | **61** | **53** |
|  | Fatty acid biosynthesis | 10 | 9 |
|  | Fatty acid degradation | 14 | 11 |
|  | Synthesis and degradation of ketone bodies | 4 | 3 |
|  | Bile acid biosynthesis | 2 | 1 |
|  | Glycerolipid metabolism | 10 | 10 |
|  | Glycerolipid metabolism | 11 | 11 |
|  | Ether lipid metabolism | 2 | 1 |
|  | alpha-Linolenic acid metabolism | 2 | 2 |
|  | Biosynthesis of unsaturated fatty acids | 6 | 5 |
|  | **Nucleotide metabolism** | **109** | **73** |
|  | Purine metabolism | 64 | 45 |
|  | Pyrimidine metabolism | 45 | 28 |
|  | **Amino acid metabolism** | **291** | **232** |
|  | Alanine, aspartate and glutamate metabolism | 27 | 21 |
|  | Glycine, serine and threonine metabolism | 39 | 32 |
|  | Cysteine and methionine metabolism | 27 | 20 |
|  | Valine, leucine and isoleucine degradation | 23 | 23 |
|  | Valine, leucine and isoleucine biosynthesis | 11 | 9 |
|  | Lysine biosynthesis | 13 | 8 |
|  | Lysine degradation | 10 | 10 |
|  | Arginine biosynthesis | 21 | 18 |
|  | Arginine and proline metabolism | 28 | 23 |
|  | Histidine metabolism | 19 | 12 |
|  | Tyrosine metabolism | 16 | 8 |
|  | Phenylalanine metabolism | 25 | 19 |
|  | Tryptophan metabolism | 13 | 11 |
|  | Phenylalanine, tyrosine and tryptophan biosynthesis | 19 | 18 |
|  | **Metabolism of other amino acids** | **39** | **42** |
|  | betaAlanine metabolism | 13 | 12 |
|  | Selenocompound metabolism | 11 | 11 |
|  | Cyanoamino acid metabolism | 6 | 5 |
|  | D-Glutamine and D-glutamate metabolism | 5 | 3 |
|  | D-Arginine and D-ornithine metabolism | 2 | 1 |
|  | D-Alanine metabolism | 2 | 2 |
|  | Glutathione metabolism | 10 | 8 |
|  | **Glycan biosynthesis and metabolism** | **30** | **24** |
|  | NGlycan biosynthesis | 1 | 1 |
|  | Various types of Nglycan biosynthesis | 1 | 1 |
|  | Glycosaminoglycan degradation | 2 | 2 |
|  | Lipopolysaccharide biosynthesis | 4 | 4 |
|  | Peptidoglycan biosynthesis | 15 | 11 |
|  | Other glycan degradation | 7 | 5 |
|  | **Metabolism of cofactors and vitamins** | **151** | **120** |
|  | Thiamine metabolism | 11 | 6 |
|  | Riboflavin metabolism | 8 | 5 |
|  | Vitamin B6 metabolism | 6 | 2 |
|  | Nicotinate and nicotinamide metabolism | 19 | 14 |
|  | Pantothenate and CoA biosynthesis | 17 | 11 |
|  | Biotin metabolism | 9 | 7 |
|  | Lipoic acid metabolism | 3 | 1 |
|  | Folate biosynthesis | 16 | 9 |
|  | One carbon pool by folate | 13 | 11 |
|  | Retinol metabolism | 2 | 23 |
|  | Porphyrin and chlorophyll metabolism | 34 | 23 |
|  | Ubiquinone and other terpenoidquinone biosynthesis | 13 | 8 |
|  | **Metabolism of terpenoids and polyketides** | **73** | **48** |
|  | Terpenoid backbone biosynthesis | 13 | 8 |
|  | Sesquiterpenoid and triterpenoid biosynthesis | 1 | 1 |
|  | Carotenoid biosynthesis | 3 | 3 |
|  | Limonene and pinene degradation | 4 | 4 |
|  | Geraniol degradation | 6 | 4 |
|  | Type I polyketide structures | 2 | 1 |
|  | Biosynthesis of ansamycins | 9 | 6 |
|  | Biosynthesis of enediyne antibiotics | 13 | 9 |
|  | Biosynthesis of type II polyketide products | 4 | 2 |
|  | Tetracycline biosynthesis | 1 | - |
|  | Polyketide sugar unit biosynthesis | 9 | 5 |
|  | Nonribosomal peptide structures | 1 | - |
|  | Biosynthesis of siderophore group nonribosomal peptides | 1 | 1 |
|  | Biosynthesis of vancomycin group antibiotics | 6 | 4 |
|  | **Biosynthesis of other secondary metabolites** | **40** | **36** |
|  | Phenylpropanoid biosynthesis | 3 | 3 |
|  | Tropane, piperidine and pyridine alkaloid biosynthesis | 2 | 2 |
|  | Glucosinolate biosynthesis | 1 | 1 |
|  | Penicillin and cephalosporin biosynthesis | 3 | 1 |
|  | Carbapenem biosynthesis | 2 | 2 |
|  | Monobactam biosynthesis | 9 | 10 |
|  | Streptomycin biosynthesis | 8 | 7 |
|  | Neomycin, kanamycin and gentamicin biosynthesis | 1 | 1 |
|  | Acarbose and validamycin biosynthesis | 2 | 3 |
|  | Novobiocin biosynthesis | 3 | 2 |
|  | Staurosporine biosynthesis | 2 | - |
|  | Phenazine biosynthesis | 1 | 2 |
|  | Prodigiosin biosynthesis | 3 | 2 |
|  | **Xenobiotics biodegradation and metabolism** | **124** | **77** |
|  | Benzoate degradation | 26 | 19 |
|  | Aminobenzoate degradation | 9 | 7 |
|  | Fluorobenzoate degradation | 3 | 1 |
|  | Chloroalkane and chloroalkene degradation | 7 | 3 |
|  | Chlorocyclohexane and chlorobenzene degradation | 9 | 5 |
|  | Toluene degradation | 5 | 2 |
|  | Xylene degradation | 8 | 5 |
|  | Nitrotoluene degradation | 2 | 1 |
|  | Ethylbenzene degradation | 1 | 1 |
|  | Styrene degradation | 7 | 5 |
|  | Atrazine degradation | 5 | 4 |
|  | Caprolactam degradation | 7 | 6 |
|  | Dioxin degradation | 5 | 3 |
|  | Naphthalene degradation | 3 | - |
|  | Polycyclic aromatic hydrocarbon degradation | 3 | 2 |
|  | Steroid degradation | 11 | 9 |
|  | Metabolism of xenobiotics by cytochrome P450 | 2 | - |
|  | Drug metabolism cytochrome P450 | 3 | 1 |
|  | Drug metabolism other enzymes | 8 | 3 |
|  | **Chemical structure transformation maps** | **1** | **1** |
|  | Biosynthesis of terpenoids and steroids | 1 | 1 |
| **Environmental Information Processing: Membrane Transport** | |  |  |
|  | ABC transporters | 83 | 61 |
|  | Phosphotransferase system PTS | 7 | 6 |
|  | Bacterial secretion system | 13 | 8 |
| **Cellular Community - Quorum sensing** | | **41** | **36** |
| **Antimicrobial Resistance** | |  |  |
|  | Betalactam resistance | 8 | 7 |
|  | Vancomycin resistance | 11 | 6 |
|  | Cationic antimicrobial peptide CAMP resistance | 3 | 2 |
