# Supplementary material for: Comparative Genomics and Biosynthetic Potential Analysis of Two Lichen-Isolated Amycolatopsis Strains
Source: Front Microbiol. 2018 Mar 13;9:369. doi: 10.3389/fmicb.2018.00369 (PMC5859366; doi:10.3389/fmicb.2018.00369)
Supplement: Supplementary file 6 [file Table3.DOCX]

Supplementary Material

Comparative Genomics and Biosynthetic Potential Analysis of Two Lichen-Isolated *Amycolatopsis* Strains

**Marina Sánchez-Hidalgo, Ignacio González, Cristian Díaz-Muñoz, Germán Martínez, Olga Genilloud***

*** Correspondence:** Olga Genilloud: olga.genilloud@medinaandalucia.es

# Supplementary Table 3: Biosynthetic gene clusters predicted by antiSMASH in the genomes of strains CA-126428 (A) and CA-128772 (B). The percentage of homology with known BGCs is indicated, as well as the MIBiG reference.

| 1. ***Amycolatopsis* sp. CA-126428** | | | |
| --- | --- | --- | --- |
| **Cluster** | **Type** | **Most similar known cluster** | **MIBiG BGC-ID** |
| Cluster 1 | Cf_putative | Plipastatin (15% of genes show similarity) | BGC0000407_c1 |
| Cluster 2 | Cf_putative | Lankacidin (20% of genes show similarity) | BGC0001100_c1 |
| Cluster 3 | Oligosaccharide-T1PKS | Aculeximycin (50% of genes show similarity) | BGC0000002_c1 |
| Cluster 4 | Cf_putative | Kosinostatin (3% of genes show similarity) | BGC0001073_c1 |
| Cluster 5 | Cf_putative | Tetarimycin (8% of genes show similarity) | BGC0000274_c1 |
| Cluster 6 | Cf_putative | - | - |
| Cluster 7 | Cf_putative | Thiolactomycin (100% of genes show similarity) | BGC0001353_c2 |
| Cluster 8 | Cf_putative | Hedamycin (6% of genes show similarity) | BGC0000233_c1 |
| Cluster 9 | Ectoine | Ectoine (100% of genes show similarity) | BGC0000853_c1 |
| Cluster 10 | T1PKS | - | - |
| Cluster 11 | Cf_putative | Phosphonoglycans (6% of genes show similarity) | BGC0000807_c1 |
| Cluster 12 | Cf_putative | - | - |
| Cluster 13 | T1PKS | - | - |
| Cluster 14 | Cf_putative | Azinomycin_B (4% of genes show similarity) | BGC0000960_c1 |
| Cluster 15 | Cf_putative | - | - |
| Cluster 16 | Cf_fatty_acid | - | - |
| Cluster 17 | Cf_putative | - | - |
| Cluster 18 | Bacteriocin | - | - |
| Cluster 19 | T1PKS | Piericidin_A1 (50% of genes show similarity) | BGC0001169_c1 |
| Cluster 20 | Cf_saccharide | Kinamycin (5% of genes show similarity) | BGC0000236_c1 |
| Cluster 21 | Cf_putative | Teicoplanin (9% of genes show similarity) | BGC0000440_c1 |
| Cluster 22 | Cf_putative | - | - |
| Cluster 23 | Cf_putative | - | - |
| Cluster 24 | Cf_putative | - | - |
| Cluster 25 | Cf_putative | A54145 (3% of genes show similarity) | BGC0000291_c1 |
| Cluster 26 | Cf_putative | K-252a (5% of genes show similarity) | BGC0000814_c1 |
| Cluster 27 | Cf_putative | - | - |
| Cluster 28 | Cf_putative | - | - |
| Cluster 29 | Cf_putative | Heme_D1 (12% of genes show similarity) | BGC0000906_c1 |
| Cluster 30 | Cf_saccharide | E-492_/_E-975 (20% of genes show similarity) | BGC0000049_c1 |
| Cluster 31 | Cf_saccharide | Coelimycin (8% of genes show similarity) | BGC0000038_c1 |
| Cluster 32 | T1PKS | Chalcomycin (12% of genes show similarity) | BGC0000035_c1 |
| Cluster 33 | Cf_putative | - | - |
| Cluster 34 | Cf_putative | - | - |
| Cluster 35 | Cf_putative | - | - |
| Cluster 36 | Cf_putative | Landepoxcin (33% of genes show similarity) | BGC0001202_c1 |
| Cluster 37 | Cf_putative | - | - |
| Cluster 38 | OtherKS | Daptomycin (3% of genes show similarity) | BGC0000336_c1 |
| Cluster 39 | NRPS | Mannopeptimycin (7% of genes show similarity) | BGC0000388_c1 |
| Cluster 40 | Cf_putative | - | - |
| Cluster 41 | Cf_putative | - | - |
| Cluster 42 | Cf_putative | - | - |
| Cluster 43 | Cf_putative | Rifamycin (17% of genes show similarity) | BGC0000136_c1 |
| Cluster 44 | Cf_putative | Sch47554_/_Sch47555 (3% of genes show similarity) | BGC0000268_c1 |
| Cluster 45 | Cf_putative | Tetarimycin (5% of genes show similarity) | BGC0000274_c1 |
| Cluster 46 | Cf_putative | - | - |
| Cluster 47 | OtherKS | Rifamycin (10% of genes show similarity) | BGC0000136_c1 |
| Cluster 48 | Cf_putative | - | - |
| Cluster 49 | Cf_putative | - | - |
| Cluster 50 | Indole | Streptomycin (2% of genes show similarity) | BGC0000717_c1 |
| Cluster 51 | Other | - | - |
| Cluster 52 | Cf_putative | - | - |
| Cluster 53 | Cf_putative | - | - |
| Cluster 54 | Cf_putative | - | - |
| Cluster 55 | Cf_saccharide | Clarexpoxcin (10% of genes show similarity) | BGC0001203_c1 |
| Cluster 56 | Cf_putative | - | - |
| Cluster 57 | T1PKS | - | - |
| Cluster 58 | T1PKS-OtherKS | Meilingmycin (4% of genes show similarity) | BGC0000093_c1 |
| Cluster 59 | Cf_putative | - | - |
| Cluster 60 | Cf_putative | - | - |
| Cluster 61 | Cf_putative | Kosinostatin (9% of genes show similarity) | BGC0001073_c1 |
| Cluster 62 | Cf_putative | Nocathiacin (4% of genes show similarity) | BGC0000609_c1 |
| Cluster 63 | Bacteriocin | - | - |
| Cluster 64 | T1PKS | - | - |
| Cluster 65 | Terpene | Isorenieratene (42% of genes show similarity) | BGC0000664_c1 |
| Cluster 66 | Cf_putative | - | - |
| Cluster 67 | Cf_putative | - | - |
| Cluster 68 | Cf_putative | - | - |
| Cluster 69 | Cf_putative | Pyrrolomycin (11% of genes show similarity) | BGC0000130_c1 |
| Cluster 70 | Cf_putative | Rifamycin (23% of genes show similarity) | BGC0000136_c1 |
| Cluster 71 | Cf_putative | - | - |
| Cluster 72 | Butyrolactone-Cf_fatty_acid | Actagardine (9% of genes show similarity) | BGC0000495_c1 |
| Cluster 73 | Cf_putative | - | - |
| Cluster 74 | T1PKS | - | - |
| Cluster 75 | Cf_putative | - | - |
| Cluster 76 | Cf_putative | Azicemicin (6% of genes show similarity) | BGC0000202_c1 |
| Cluster 77 | Cf_saccharide | - | - |
| Cluster 78 | Cf_putative | Fosfazinomycin (14% of genes show similarity) | BGC0000937_c1 |
| Cluster 79 | Siderophore | Macrotetrolide (33% of genes show similarity) | BGC0000244_c1 |
| Cluster 80 | T1PKS | - | - |
| Cluster 81 | Cf_putative | - | - |
| Cluster 82 | Cf_putative | - | - |
| Cluster 83 | Cf_putative | - | - |
| Cluster 84 | Cf_putative | - | - |
| Cluster 85 | Cf_putative | Tetronasin (7% of genes show similarity) | BGC0000163_c1 |
| Cluster 86 | Cf_putative | - | - |
| Cluster 87 | Cf_putative | Azicemicin (4% of genes show similarity) | BGC0000202_c1 |
| Cluster 88 | Cf_putative | - | - |
| Cluster 89 | Cf_putative | Frankiamicin (14% of genes show similarity) | BGC0001197_c1 |
| Cluster 90 | NRPS-T1PKS-TransatPKS | Taromycin (33% of genes show similarity) | BGC0000439_c1 |
| Cluster 91 | Cf_saccharide | Endophenazines (13% of genes show similarity) | BGC0000934_c1 |
| Cluster 92 | Cf_putative | Tetrocarcin_A (4% of genes show similarity) | BGC0000162_c1 |
| Cluster 93 | NRPS | Arginomycin (10% of genes show similarity) | BGC0000883_c1 |
| Cluster 94 | Cf_putative | Pimaricin (11% of genes show similarity) | BGC0000125_c1 |
| Cluster 95 | Cf_saccharide | - | - |
| Cluster 96 | Cf_fatty_acid | - | - |
| Cluster 97 | Cf_putative | Salinomycin (4% of genes show similarity) | BGC0000144_c1 |
| Cluster 98 | Cf_putative | - | - |
| Cluster 99 | Cf_putative | Nystatin (18% of genes show similarity) | BGC0000115_c1 |
| Cluster 100 | Cf_putative | Thiotetronate_Tu_3010 (5% of genes show similarity) | BGC0001352_c1 |
| Cluster 101 | Cf_putative | - | - |
| Cluster 102 | Cf_putative | - | - |
| Cluster 103 | Terpene | Actinomycin (17% of genes show similarity) | BGC0000296_c1 |
| Cluster 104 | Cf_putative | - | - |
| Cluster 105 | Terpene | Actagardine (9% of genes show similarity) | BGC0000495_c1 |
| Cluster 106 | Cf_saccharide | - | - |
| Cluster 107 | Terpene-Cf_saccharide-NRPS | WS9326 (17% of genes show similarity) | BGC0001297_c1 |
| Cluster 108 | Cf_putative | SCO-2138 (14% of genes show similarity) | BGC0000595_c1 |
| Cluster 109 | Cf_fatty_acid | Maklamicin (10% of genes show similarity) | BGC0001288_c1 |
| Cluster 110 | NRPS | Albachelin (40% of genes show similarity) | BGC0001211_c1 |
| Cluster 111 | Cf_putative | - | - |
| Cluster 112 | Cf_putative | Svaricin (6% of genes show similarity) | BGC0001382_c1 |
| Cluster 113 | Cf_putative | - | - |
| Cluster 114 | Bacteriocin | - | - |
| Cluster 115 | Cf_putative | - | - |
| Cluster 116 | Cf_putative | Rifamycin (12% of genes show similarity) | BGC0000136_c1 |
| Cluster 117 | Cf_putative | Frankiamicin (14% of genes show similarity) | BGC0001197_c1 |
| Cluster 118 | Cf_putative | - | - |
| Cluster 119 | Cf_fatty_acid | Chlorizidine_A (7% of genes show similarity) | BGC0001172_c1 |
| Cluster 120 | Cf_putative | - | - |
| Cluster 121 | Cf_putative | - | - |
| Cluster 122 | Cf_putative | - | - |
| Cluster 123 | Cf_putative | - | - |
| Cluster 124 | Cf_fatty_acid | Asukamycin (4% of genes show similarity) | BGC0000187_c1 |
| Cluster 125 | T1PKS-Nucleoside-NRPS | Cremimycin (20% of genes show similarity) | BGC0000042_c1 |
| Cluster 126 | T1PKS | Sporolide (53% of genes show similarity) | BGC0000150_c1 |
| Cluster 127 | Cf_saccharide | Meilingmycin (2% of genes show similarity) | BGC0000093_c1 |
| Cluster 128 | OtherKS | - | - |
| Cluster 129 | Lantipeptide | Paromomycin (5% of genes show similarity) | BGC0000712_c1 |
| Cluster 130 | Cf_putative | Mycinamicin (9% of genes show similarity) | BGC0000102_c1 |
| Cluster 131 | Cf_putative | - | - |
| Cluster 132 | Butyrolactone | - | - |
| Cluster 133 | NRPS | Stenothricin (9% of genes show similarity) | BGC0000431_c1 |
| Cluster 134 | Lantipeptide | - | - |
| Cluster 135 | Cf_putative | - | - |
| Cluster 136 | Cf_putative | Neocarzinostatin (4% of genes show similarity) | BGC0000112_c1 |
| Cluster 137 | Cf_saccharide | Caprazamycin (28% of genes show similarity) | BGC0000875_c2 |
| Cluster 138 | T1PKS | Concanamycin_A (35% of genes show similarity) | BGC0000040_c1 |
| Cluster 139 | Cf_putative | Allylmalonyl-CoA (20% of genes show similarity) | BGC0000886_c1 |
| Cluster 140 | Cf_putative | Frankiamicin (14% of genes show similarity) | BGC0001197_c1 |
| 1. ***Amycolatopsis* sp. CA-128772** | | | |
| **Cluster** | **Type** | **Most similar known cluster** | **MIBiG BGC-ID** |
| Cluster 1 | Other | Herboxidiene (2% of genes show similarity) | BGC0001065_c1 |
| Cluster 2 | Cf_putative | - | - |
| Cluster 3 | Cf_putative | - | - |
| Cluster 4 | Cf_putative | Bafilomycin (33% of genes show similarity) | BGC0000028_c1 |
| Cluster 5 | Cf_putative | Reveromycin (6% of genes show similarity) | BGC0000135_c1 |
| Cluster 6 | Cf_putative | - | - |
| Cluster 7 | Cf_putative | - | - |
| Cluster 8 | Cf_putative | - | - |
| Cluster 9 | Indole | Streptomycin (2% of genes show similarity) | BGC0000717_c1 |
| Cluster 10 | Cf_putative | - | - |
| Cluster 11 | Cf_putative | Allylmalonyl-CoA (20% of genes show similarity) | BGC0000886_c1 |
| Cluster 12 | Cf_saccharide | Arginomycin (10% of genes show similarity) | BGC0000883_c1 |
| Cluster 13 | Cf_putative | - | - |
| Cluster 14 | Cf_putative | - | - |
| Cluster 15 | Cf_putative | Plipastatin (15% of genes show similarity) | BGC0000407_c1 |
| Cluster 16 | Cf_saccharide | - | - |
| Cluster 17 | NRPS-T1PKS | ECO-02301 (39% of genes show similarity) | BGC0000052_c1 |
| Cluster 18 | T1PKS | Hitachimycin (22% of genes show similarity) | BGC0001194_c1 |
| Cluster 19 | Cf_saccharide | - | - |
| Cluster 20 | Cf_putative | - | - |
| Cluster 21 | Cf_putative | - | - |
| Cluster 22 | Cf_putative | - | - |
| Cluster 23 | Cf_saccharide | Lividomycin (10% of genes show similarity) | BGC0000708_c1 |
| Cluster 24 | Cf_putative | - | - |
| Cluster 25 | Butyrolactone | - | - |
| Cluster 26 | Cf_putative | - | - |
| Cluster 27 | Terpene-Cf_fatty_acid -NRPS | WS9326 (17% of genes show similarity) | BGC0001297_c1 |
| Cluster 28 | Ladderane-Cf_fatty_acid -NRPS | WS9326 (17% of genes show similarity) | BGC0001297_c1 |
| Cluster 29 | Cf_saccharide-Cf_fatty_acid | - | - |
| Cluster 30 | T1PKS-NRPS-Lantipeptide | Kedarcidin (3% of genes show similarity) | BGC0000081_c1 |
| Cluster 31 | Cf_putative | - | - |
| Cluster 32 | T1PKS | Sanglifehrin_A (11% of genes show similarity) | BGC0001042_c1 |
| Cluster 33 | NRPS | Galbonolides (6% of genes show similarity) | BGC0000065_c1 |
| Cluster 34 | Cf_saccharide | Mycinamicin (9% of genes show similarity) | BGC0000102_c1 |
| Cluster 35 | Lantipeptide | - | - |
| Cluster 36 | NRPS-Cf_saccharide-Amglyccycl | Acarbose (10% of genes show similarity) | BGC0000691_c1 |
| Cluster 37 | Cf_putative | - | - |
| Cluster 38 | Cf_putative | - | - |
| Cluster 39 | Cf_putative | - | - |
| Cluster 40 | Cf_saccharide | Macrotetrolide (33% of genes show similarity) | BGC0000244_c1 |
| Cluster 41 | Cf_putative | - | - |
| Cluster 42 | Bacteriocin | - | - |
| Cluster 43 | Cf_putative | - | - |
| Cluster 44 | Siderophore | - | - |
| Cluster 45 | T1PKS | Rifamycin (54% of genes show similarity) | BGC0000136_c1 |
| Cluster 46 | Cf_saccharide | - | - |
| Cluster 47 | Cf_putative | Frankiamicin (14% of genes show similarity) | BGC0001197_c1 |
| Cluster 48 | Cf_saccharide | A201A (6% of genes show similarity) | BGC0000873_c1 |
| Cluster 49 | Cf_putative | - | - |
| Cluster 50 | OtherKS | A54145 (3% of genes show similarity) | BGC0000291_c1 |
| Cluster 51 | Cf_fatty_acid | Chlorizidine_A (7% of genes show similarity) | BGC0001172_c1 |
| Cluster 52 | Bacteriocin | - | - |
| Cluster 53 | Ectoine | Ectoine (100% of genes show similarity) | BGC0000853_c1 |
| Cluster 54 | Terpene | 2-methylisoborneol (100% of genes show similarity) | BGC0000657_c1 |
| Cluster 55 | Cf_saccharide | - | - |
| Cluster 56 | Cf_putative | - | - |
| Cluster 57 | Cf_putative | Rifamycin (10% of genes show similarity) | BGC0000136_c1 |
| Cluster 58 | Cf_putative | Frankiamicin (14% of genes show similarity) | BGC0001197_c1 |
| Cluster 59 | Cf_putative | - | - |
| Cluster 60 | Cf_putative | - | - |
| Cluster 61 | Cf_saccharide | Coelimycin (8% of genes show similarity) | BGC0000038_c1 |
| Cluster 62 | Cf_saccharide | - | - |
| Cluster 63 | Cf_putative | - | - |
| Cluster 64 | Cf_saccharide | - | - |
| Cluster 65 | Cf_putative | - | - |
| Cluster 66 | Cf_putative | Landepoxcin (27% of genes show similarity) | BGC0001202_c1 |
| Cluster 67 | Cf_putative | - | - |
| Cluster 68 | Cf_putative | - | - |
| Cluster 69 | Cf_putative | - | - |
| Cluster 70 | Cf_saccharide | - | - |
| Cluster 71 | NRPS | Mannopeptimycin (7% of genes show similarity) | BGC0000388_c1 |
| Cluster 72 | Cf_putative | - | - |
| Cluster 73 | Cf_putative | - | - |
| Cluster 74 | Cf_saccharide | - | - |
| Cluster 75 | T1PKS | Kedarcidin (13% of genes show similarity) | BGC0000081_c1 |
| Cluster 76 | Oligosaccharide-T1PKS-NRPS | Maduropeptin (22% of genes show similarity) | BGC0001008_c1 |
| Cluster 77 | Cf_putative | - | - |
| Cluster 78 | Cf_putative | - | - |
| Cluster 79 | Cf_saccharide-NRPS-T1PKS | Daptomycin (6% of genes show similarity) | BGC0000336_c1 |
| Cluster 80 | Cf_putative | - | - |
| Cluster 81 | Cf_saccharide | - | - |
| Cluster 82 | Cf_putative | Nystatin (9% of genes show similarity) | BGC0000115_c1 |
| Cluster 83 | Cf_putative | - | - |
| Cluster 84 | Cf_putative | - | - |
| Cluster 85 | Cf_putative | - | - |
| Cluster 86 | Cf_saccharide | Caprazamycin (28% of genes show similarity) | BGC0000875_c2 |
| Cluster 87 | Cf_saccharide | - | - |
| Cluster 88 | Cf_saccharide | SCO-2138 (14% of genes show similarity) | BGC0000595_c1 |
| Cluster 89 | Cf_putative | - | - |
| Cluster 90 | T1PKS | - | - |
| Cluster 91 | Cf_putative | - | - |
| Cluster 92 | Cf_putative | - | - |
| Cluster 93 | Cf_putative | - | - |
| Cluster 94 | Cf_putative | - | - |
| Cluster 95 | Cf_putative | - | - |
| Cluster 96 | Cf_putative | A40926 (3% of genes show similarity) | BGC0000289_c1 |
| Cluster 97 | Cf_putative | Meilingmycin (2% of genes show similarity) | BGC0000093_c1 |
| Cluster 98 | Cf_putative | Polyoxypeptin (8% of genes show similarity) | BGC0001036_c1 |
| Cluster 99 | Cf_putative | - | - |
| Cluster 100 | Cf_putative | - | - |
| Cluster 101 | Cf_putative | - | - |
| Cluster 102 | Terpene | Fluorometabolite (33% of genes show similarity) | BGC0000903_c1 |
| Cluster 103 | Cf_putative | - | - |
| Cluster 104 | Cf_putative | - | - |
| Cluster 105 | Cf_saccharide-T1PKS | - | - |
| Cluster 106 | OtherKS-T1PKS | Meilingmycin (3% of genes show similarity) | BGC0000093_c1 |
| Cluster 107 | NRPS | Scabichelin (90% of genes show similarity) | BGC0000423_c1 |
| Cluster 108 | Cf_saccharide-NRPS-T1PKS | Nostopeptolide (25% of genes show similarity) | BGC0001028_c1 |
| Cluster 109 | T1PKS | Tallysomycin (5% of genes show similarity) | BGC0001048_c1 |
| Cluster 110 | Cf_putative | - | - |
| Cluster 111 | Cf_saccharide | Arginomycin (10% of genes show similarity) | BGC0000883_c1 |
| Cluster 112 | OtherKS | - | - |
| Cluster 113 | Cf_putative | Azinomycin_B (4% of genes show similarity) | BGC0000960_c1 |
| Cluster 114 | Lassopeptide | SF2575 (6% of genes show similarity) | BGC0000269_c1 |
| Cluster 115 | Cf_saccharide | Roseoflavin (50% of genes show similarity) | BGC0000927_c1 |
| Cluster 116 | Cf_saccharide | Esmeraldin (8% of genes show similarity) | BGC0000935_c1 |
| Cluster 117 | Cf_putative | - | - |
| Cluster 118 | Lantipeptide | - | - |
| Cluster 119 | Cf_putative | Salinomycin (4% of genes show similarity) | BGC0000144_c1 |
| Cluster 120 | Cf_fatty_acid | - | - |
| Cluster 121 | Cf_saccharide | - | - |
| Cluster 122 | Cf_saccharide | - | - |
| Cluster 123 | Cf_saccharide | - | - |
| Cluster 124 | Cf_saccharide | Meilingmycin (2% of genes show similarity) | BGC0000093_c1 |
| Cluster 125 | Cf_saccharide | Kinamycin (5% of genes show similarity) | BGC0000236_c1 |
| Cluster 126 | Cf_saccharide | - | - |
| Cluster 127 | Cf_putative | - | - |
| Cluster 128 | Cf_putative | - | - |
| Cluster 129 | Cf_putative | - | - |
| Cluster 130 | Cf_fatty_acid | Asukamycin (3% of genes show similarity) | BGC0000187_c1 |
| Cluster 131 | Cf_putative | - | - |
| Cluster 132 | Cf_putative | - | - |
| Cluster 133 | Cf_saccharide | - | - |
| Cluster 134 | T1PKS | Amphotericin (47% of genes show similarity) | BGC0000015_c1 |
| Cluster 135 | T1PKS | - | - |
| Cluster 136 | T1PKS | Indanomycin (21% of genes show similarity) | BGC0000079_c1 |
| Cluster 137 | Cf_saccharide | - | - |
| Cluster 138 | Cf_putative | - | - |
| Cluster 139 | Cf_putative | - | - |
| Cluster 140 | Cf_putative | - | - |
